# Root phenotypic detection of different vigorous maize seeds based on Progressive Corrosion Joining algorithm of image

**DOI:** 10.1186/s13007-019-0518-5

**Published:** 2019-11-18

**Authors:** Wei Lu, Ye Li, Yiming Deng

**Affiliations:** 1 College of Engineering, Nanjing Agricultural University, Nanjing, 210031 China; 2 Robot Sensor and Control Technology Laboratory, Nanjing, 210031 China; 3 NDE Laboratory, Electrical and Computer Engineering, Michigan State University, East Lansing, 48824 USA

**Keywords:** Root phenotyping, Progressive Corrosion Joining, Seed vigor

## Abstract

**Background:**

The root phenotypes of different vigorous maize seeds vary a lot. Imaging roots of growing maize is a non-invasive, affordable and high throughput approach. However, it’s difficult to get integral root images because of the block of the soil. The paper proposed an algorithm to repair incomplete root images for maize root fast non-invasive phenotyping detection.

**Results:**

A two-layer transparent stress growth device with two concentric cylinders was developed as mesocosms and the maize seeds were planted in the annulus of it. The maize roots grow in soil against two acrylic plastic surfaces due to the press of the small growing area to acquire more root details during roots visualization and imaging. Even though, parts of the roots are occluded which means that it’s tough to extract the information of root general physical construction. For recovering gaps from disconnected root segments, Progressive Corrosion Joining (PCJ) algorithm was proposed based on the physiological characteristics of hydrotropism, geostrophic and continuity with three steps which are root image thinning, progressive corrosion and joining processing respectively. The experiments indicate that maize phenotyping parameters are negative correlation with seed aging days. And specifically, Root Number (RTN), Root Length (RTL), Root Width (RTW) and Root Extension Length (REL) of unaged and 14-day-aged maize seeds are decreased from 15.40, 82.40 mm, 1.53 mm and 82.20 mm to 4.58, 38.6 mm, 1.35 mm and 55.20 mm, and the growing speed of them are changed from 1.68 per day, 8.80 mm/d, 0.06 mm/d, 9.0 mm/d to 0.70 per day, 4.3 mm/d, 0.05 mm/d and 5.70 mm/d respectively. Whereas Root Extension Angle (REA) is basically irrelevant with the level of maize seed aging.

**Conclusion:**

The developed double-layer Annular Root Phenotyping Container (ARPC) can satisfy the general physical construction of maize as well as push each root growing along the inner wall of the container which help to acquire more root information. The presented novel PCJ algorithm can recover the missing parts, even for big gaps, of maize roots effectively according to root morphological properties. The experiments show that the proposed method can be applied to evaluate the vigor of maize seeds which has vast application prospect in high throughput root phenotyping area.

## Background

Plant root is an important organ for water, nutrients and minerals uptake. Root morphology research is an important content of plant nutrition, physiology, breeding and ecology [1, 2]. As the largest production crop in the world, maize can be used as both food and fodder whose root, as the plant holder, greatly affects the ability of press resistance such as water and wind which significantly results in final yield reduction. Therefore, root phenotyping parameters of maize seeding such as root spatial distribution, root length, root width, root extension characteristics, root hair density and bending angle are applied to evaluate maize seed vigor.

For root character observation clearly and directly, germination paper method was proposed to seed screening such as soybean [3], corn [4], wheat [5], rapeseed [6] and pearl millet [7]. Computer vision technology was introduced to speed up germination paper detection but the root can only grow on the surface of paper [8]. To extend plant root grow in 3D space from 2D surface, hydroponics [9] and atomization cultivation [10] patterns were used but root morphology is affected by gravity. So, the gel transparent matrix which can keep the plant roots in a consistent spatial structure has been developed, whereas the chemical properties of the matrix are different from that of soil which may affect root development and configuration [11]. The research mentioned above can overcome the problem of soil interference, control the homogeneity of the culture medium and minimize the inherent variability of the observed root traits as well as enable clear imaging. However, plant root system growing environment in the field is essentially different from that of air, water and matrix.

Therefore, it becomes a key problem to obtain as much root information as possible on the premise of ensuring the root growth in the soil. The most direct and effective traditional approach such as soil core, soil column and root canal [12, 13] were applied to study a root system by digging out the root and observe it by manual inspecting, machine vision [14], spectral imaging [15] and laser scanning imaging [16] technologies after removing the soil. However, it will cause irreversible damage and configurational change to the root system. To achieve non-destructive in situ plant root phenotypic detection, X-ray computed tomography (CT) [17, 18] and nuclear magnetic resonance (NMR) imaging technology [19] used in medicine science popularly were introduced to obtain clear stereoscopic images of roots, but they hurt the roots to some extent after repeated measurements and the instruments are also very expensive.

Minirhizotron [20, 21] and its improvement methods [22], also as a non-destructive way, were provided to monitor plant roots over a long period of time in the field for forests, orchards and agro-ecosystems research [23–26] but are labor intensive and time consuming, even more serious is that the methods can only sample local pictures of roots and lead to excessive root reproduction because the set can disturb the balance of roots and soil.

For obtaining root general and detailed information in the soil rapidly, non-destructively and simultaneously, root phenotype pipeline system [27] was developed to image plant roots in cylindrical or flat-plate type transparent culture containers which is popularly used in breeding industry nowadays, whereas parts of root are occluded by soil which results in incomplete root image even under perfect root segmentation. So, root gap correcting algorithm was proposed for the extraction of finely grained root system architecture traits using deep learning CNN and obtained good effects but the training set and testing set are idealized manual drawn pictures which need a lot of manual work [28–30].

In this paper, we developed a double-layer Annular Root Phenotyping Container (ARPC) to acquire more root information considering the spatial configuration of maize root. Five rules were given for maize root gaps recovering considering the physiological characteristics of hydrotropism, geostrophic and continuity to propose a novel maize root gaps recovering algorithm, Progressive Corrosion Joining (PCJ) algorithm. Finally, the proposed algorithm was applied to improve maize root images which can be used to evaluate the vigor of different aging-day seeds effectively. The proposed method, compared with other existed root phenotyping approaches, can be used for high-throughput, low-cost, non-destructive phenotypic detection of maize roots, and can obtain more phenotypic information of maize roots. So, the proposed ARPC combing with PCJ algorithm is a good method which has vast application prospect in high throughput root phenotyping area (Tables 1, 2).

**Table 1:** The captures of the cylinder

Serial number	Height (cm)	Radius (cm)	Wall thickness (mm)	Material
Cylinder 1	15	6	2	Acrylic sheet
Cylinder 2	15	5	2	Acrylic sheet

**Table 2:** The captures of the ring

Outer diameter (cm)	Inner radius (cm)	Number of through holes	Through hole diameter (cm)	Thickness (mm)	Material
6	5	8	0.8	4	Acrylic sheet

## Materials and methods

### Plant culture device and seed growth conditions

**Fig. 1:**
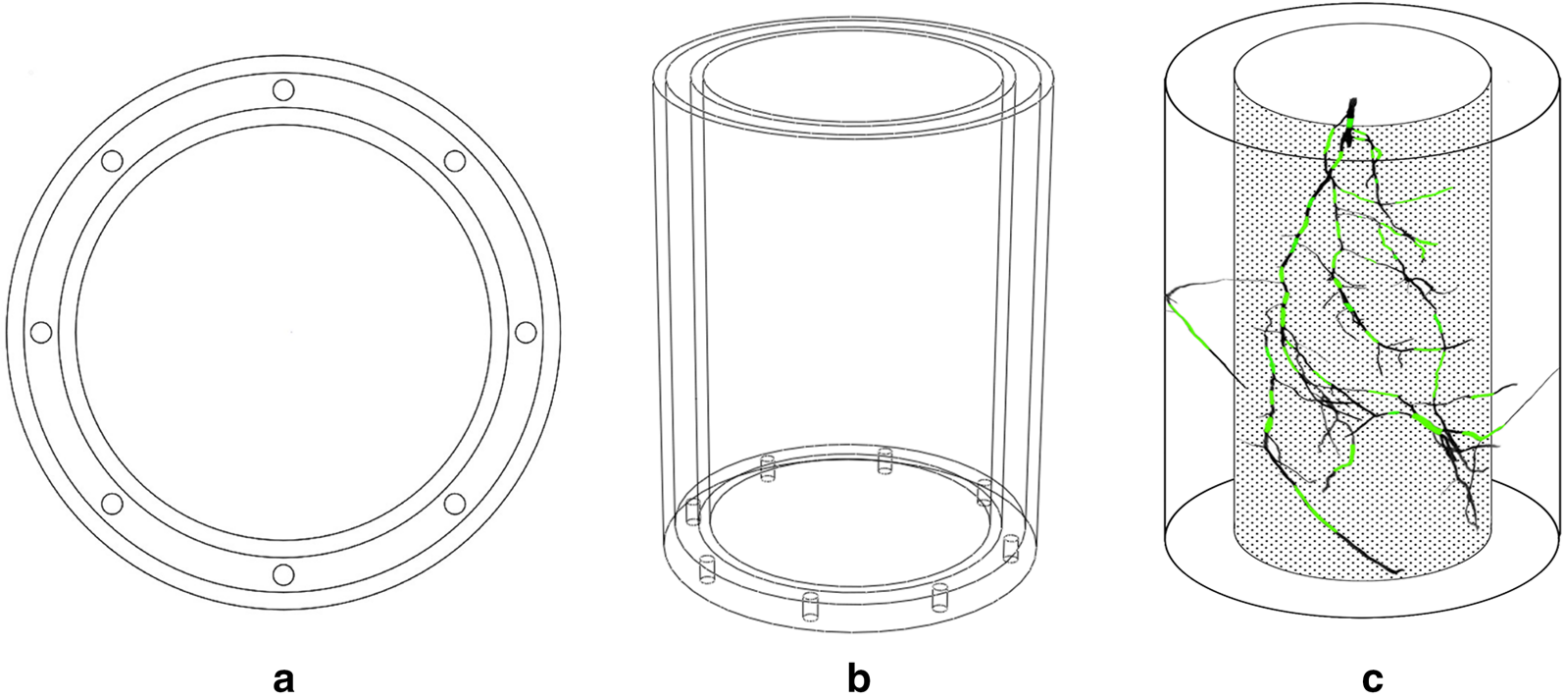
The double-layer Annular Root Phenotyping Container. **a** is the vertical view of ARPC. **b** is the front view of ARPC. **c** is the simulation view, and the green part of the root can be seen by the naked eye

Because of the root growing in straight lines and its tendency such as hydrotropism, geostrophic, a double-layer ARPC device (Fig. 1) was developed to get more root information. The APRC device consists of two concentric hollow acrylic cylinders of different diameters with the same height and a ring with 8 leaking holes. The hollow annular between two acrylic layers are filled with homogeneous cultivated soil (organic matter 478 g/1000 g, N + P+K: 6.75 mg/1000 g, probiotics: 90 μg/1000 g, trace element 1.7 μg/1000 g, humic acid: 130 μg/1000 g, pH 6.5–6.8). *Heinuo*-*1* (Black Corn, Shangdu Seed Co., Ltd, Henan, China) maize seeds were sown in the annular in the greenhouse in Nanjing Agricultural University, College of Engineering (32° 18′ N, 118° 46′ E). The parameters of the device are shown in Fig. 1.

### Image acquisition device

The major factor influencing image processing quality is the reflection from the device. To obtain high quality pictures, the following devices are used.

**Fig. 2:**
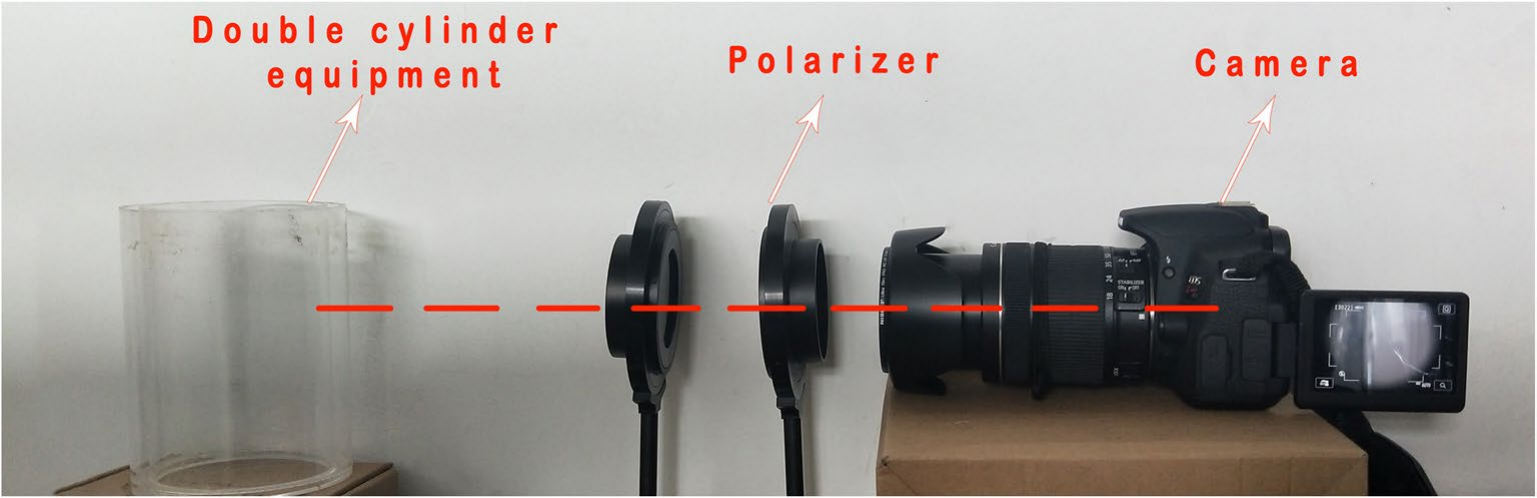
Shooting scene pictures

1. Camera: Canon D600 digital SLR camera (18–135 mm medium focal length lens, Canon). The camera was set to macro mode. The shutter speed, aperture and sensor sensitivity were set as 1/3 s, 5 mm and 800 ISO respectively.
2. Polarizer: A set of polarizers were placed in order along the axis of light to eliminate reflection from the plant container. The angle of polarization between the polarizer and analyzer was adjusted manually to optimum condition.
3. The centers of camera lens, polarizer, analyzer and root were set along the same light axis. The lens of camera focused on the root center to acquire clear root images (Fig. 2).

### Experimental procedure

1. Seed aging: The screened seeds were divided into 8 parts after dormancy treatment and were artificially aged for 0, 2, 4, 6, 8, 10, 12 and 14 days respectively. During aging time,seeds were placed in glass trays and enclosed in intelligent artificial climate chamber (RXZ type, Ningbo Jiangnan Instrument Factory) under the condition of 45 °C and 90% humidity.
2. Seed disinfection: Black corn *Heinuo*-*1* seeds with the germination not less than 85% were used for root phenotyping experiment. The seeds were immersed in 70% (v/v) ethanol and 5% sodium hypochlorite solution for 30 s and 10 min respectively before transferred into ARPC device.
3. Seeding culture: Two seeds were sowed in each ARPC device and the seedlings were poured once every 5 days with 5 ml volume at 9 am after seeds sowing. The two sides of ARPC device were covered with black plastic film to simulate root growing luminous environment in the field (Fig. 3).

**Fig. 3:**
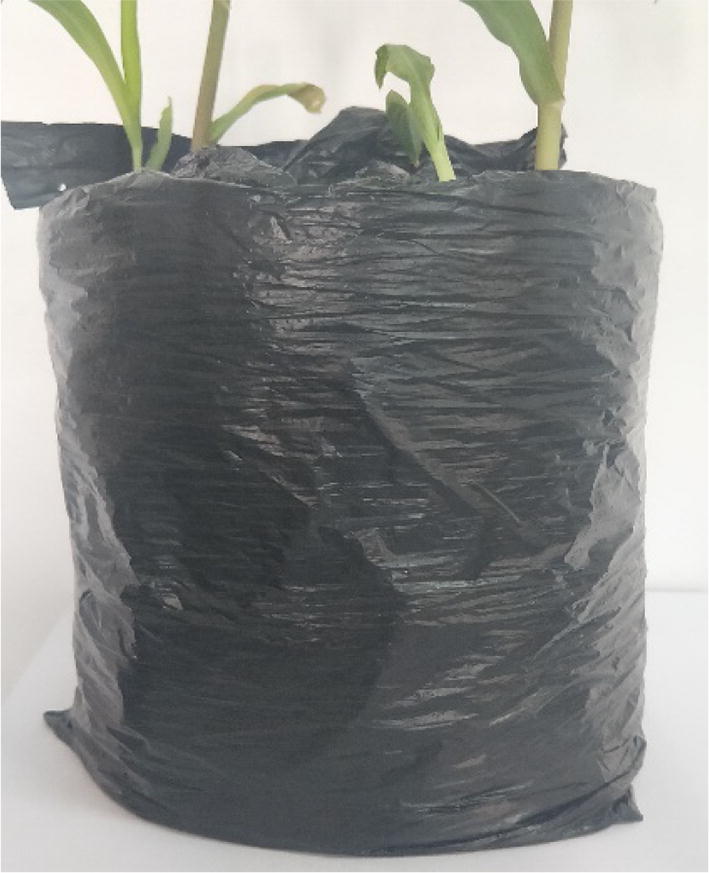
The picture of shaded device
4. Maize root images acquisition: Six seeds of every aging day were used for experiment, as a total 48 seedings were used for root imaging. Root images were collected indoor twice every day at 9 am and 4 pm for monitoring the development of seeding roots dynamically, and 4 pictures with different perspectives (covering 360°) were acquired every time. Each culture container was fixed on the same marked position and rotated manually while imaging to get the sequence of images cover 360° views.

## Root repair

### Pre-processing

**Fig. 4:**
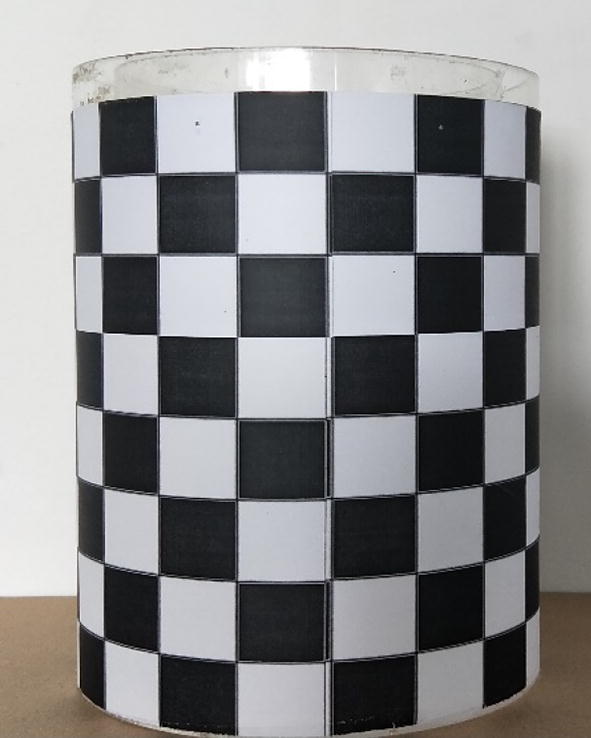
The device pasted with the checkered paper for correcting
- The transformational approaches such as gray, binary, median filtering, gaussian filtering and flooding algorithms were used successively as preprocessing to obtain clear images from image noise made by light reflection and soil color difference.

- Image stitching: The paper judges the continuity of maize root by detecting whether there are more than 4 consecutive white pixels at the image edge. And the feature points between the adjacent images are extracted for stitching a complete root according to the L-ORB algorithm if the edge of root image is incomplete [31].
- Image calibration: Considering that the images taken by the camera are distorted, a piece of checkerboard paper was pasted to the surface of the ARPC for camera calibration with reference to the literature [32] (Fig. 4).

### Feature point extraction

Two rules were proposed to determine root feature points as below before image processing algorithm.

- Endpoint decision rule: One endpoint only connects and belongs to one root. Define the image area as *Ω*, *P* is one of the point on it. The minimized square *D* centered at point *P* with 9 pixels is selected to judge whether point *P* connects with no more than 2 connected points. The mathematical expression of the rule is as follows.
  1 $$ e\_p = \left\{ {\begin{aligned} & {Ture,\;\;n = 1} \\ &{Ture,\;\;(n = 2)\;and\;(Q\;{\text{are}}\;connected)} \\ &{False,\;\;else} \\ \end{aligned} } \right. $$
  *m* is 8 points in square *D* except point *P*, point *Q* $\in$ *m*, *n* is the number of points in *m*. *e_p* is the *end point*.
- Bifurcation point rule: The branching point is connected to multiple roots. Define the image area as *Ω*, *P* is one of the point on it. The minimized square *D* centered at point *P* with 9 pixels is selected to judge whether point *P* connects with not less than 2 disconnected points. The mathematical expression of the rule is as follows.
  2 $$ b\_p = \left\{ \begin{aligned} & Ture,\;\;(n > 1)\;and\;(Q\;{\text{are}}\;connected) \hfill \\ & False,\;\;else \hfill  \\  \end{aligned} \right. $$
  *m* is 8 points in square *D* except point *P*, point *Q* $\in$ *m*, *n* is the number of points in *m*. *b_p* is the *bifurcation point*.

**Fig. 5:**
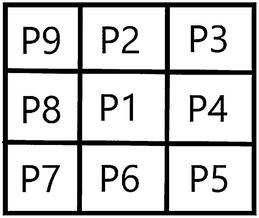
Foreground pixel illustration

**Fig. 6:**
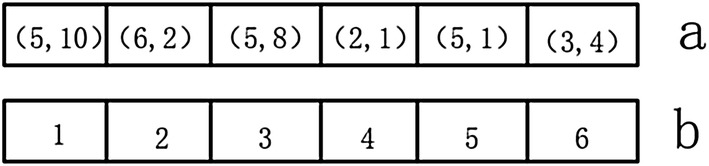
A queue for dynamically storing endpoints information
- Step 3: Image mapping
  Create an empty image *“c”* with the same size of thinning image, then save the elements of *“b”* in the corresponding coordinates of *“c”*, that is, the coordinates of the elements in *“c”* are equal to the values of *“a”*.
  5 $$ c(a[i]) = b[index(a[i])] $$
  where *index* is the coordinate of the index value of a queue.
- Step 4: Progressive corrosion

Based on the above two rules, the Progressive Corrosion Joining algorithm is presented as follow steps:

- Step 1: Image thinning
  Zhang-Suen thinning algorithm was used to refine the root trunk [33]. Firstly, all the foreground pixels are traversed and removed if they meet the following conditions (Fig. 5).

  3 $$ \left\{ \begin{array}{ll}  2 \le N(P1) \le 6 \hfill  \\ S(P1)  = 1 \hfill & \\ P2*P4*P6 = 0 \hfill & \\ P4*P6*P8 = 0 \hfill  \end{array} \right. $$
  where *N(P1)* represents the number of foreground pixel points among the 8 pixels adjacent to *P1*; *S(P1)* represents the cumulative numbers of 0–1 model, the adjacent two pixels are 0 and 1, counting along *P2*-*P9*-*P2* line, where 0 represents the background and 1 indicates the prospect.
  Then, *P1* is also traversed and removed if it satisfies the conditions below.
  4 $$ \left\{ \begin{array}{ll} 2 \le N(P1) \le 6 \hfill \\ S(P1) = 1 \hfill \\ P2*P4*P8 = 0 \hfill \\ P2*P6*P8 = 0 \hfill \\ \end{array} \right. $$
  The refined root will be obtained after the above two steps.
- Step 2: Endpoint extraction
  The endpoint set *M* is created by using the endpoint decision rule. The coordinates of all the elements in *M* are stored in the queue “*a”*, the elements in *M* are numbered and the serial numbers are stored in queue *“b”*.
  To obtain the endpoint point set *M* of the refined image, and numbering the element *M[i]* in *M*, storing the element *M[i]* in the queue *“a”*, and storing the sequence number in the queue *“b”*. There are only two endpoints for each line, and after subsequent processing, the point set numbers on each line will be the same (Fig. 6).

The steps of the proposed progressive corrosion algorithm are as follow and loop until *“a”* is empty.

- A point *P* pops off from queue *“a”*, then the value of the point in the thinning image which coordinate equal to the value of *P* is set to 0.
- If one of the surrounding 8 pixels, defined as point *q*, of P in *“c”* has been assigned a serial number, then assign the value of *P* to *q*.
- If *P* is an endpoint, the surrounding point connected to *P* is named as *P1*. And the value of the point with the same coordinate with *P1* in *“c”* is assigned to the value of *P*.
- If *P* is a bifurcation point, the coordinates of the surrounding points of *P* are pushed into *“a”*. Then the surrounding points are numbered in *“b”* and the corresponding points in *“c”* are assigned the corresponding value.

**Fig. 7:**
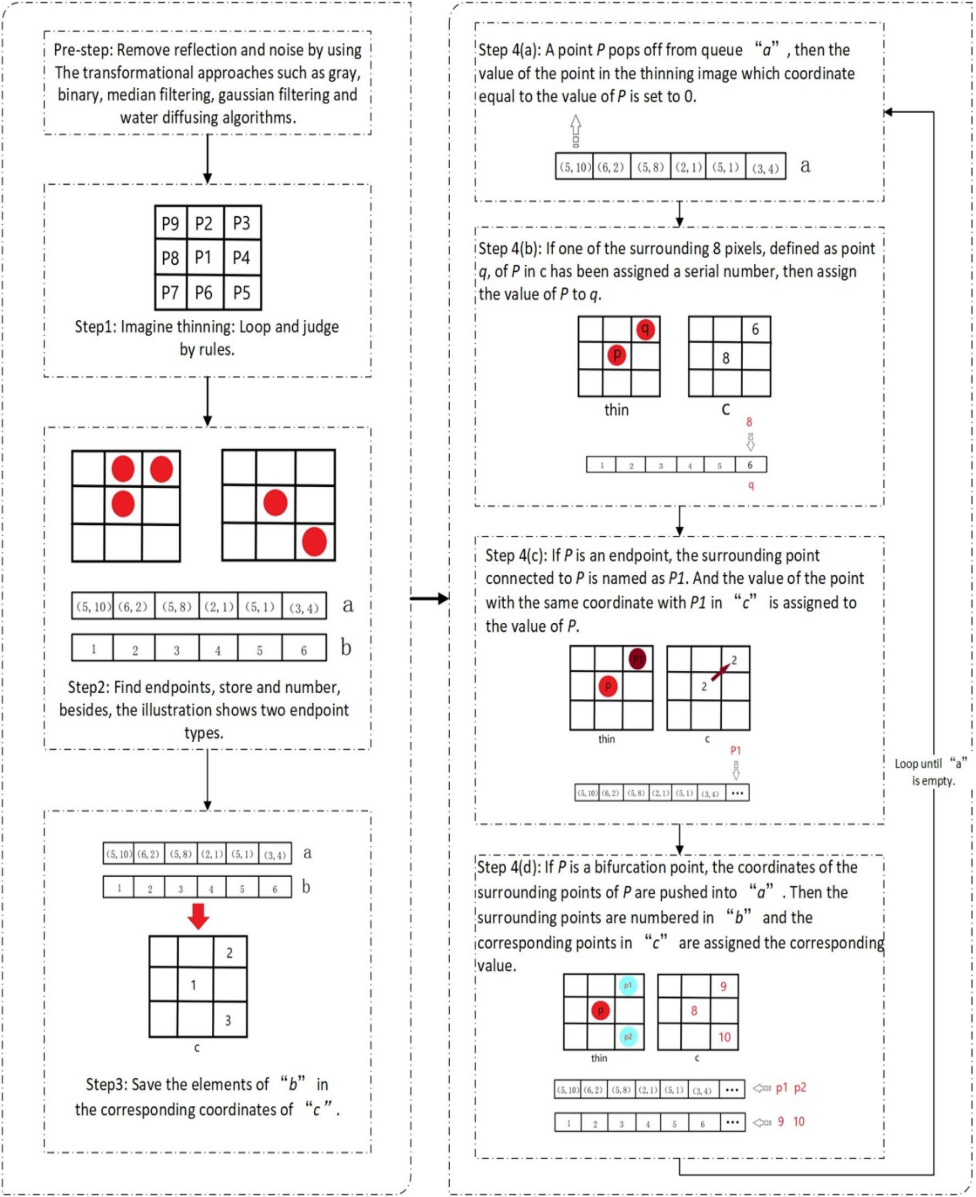
Progressive Corrosion algorithm Flowchart

The flowchart of the PCJ algorithm can be seen at Fig. 7.

## Root connection

After the operation of above steps, each continuous segment of a root has the same serial number. Three rules were proposed for root gap connection.

### Internal continuity rule

The pixels of each root segment in the thinning image are step-forced but a root should be smooth actually. So, the least squares method is used to fit the pixels as shown in the formula below.

6 $$ f(x) = a_{0} + a_{1} x + a_{2} x + \cdots + a_{k} x_{k} $$

where x and f(x) are the abscissa and ordinate respectively.

The loss value is expressed as below.

7 $$ l = \sum\limits_{i = 1}^{n} {[y_{i} - (a_{0} + a_{1} x + \cdots + a_{k} x_{k} )]^{2} } $$

*l* is loss.

The partial derivatives of *a*_*i*_ (*i *= 1…*k*) are absolved and let be 0, we can get the Vandermonde matrix,

8 $$ \left[ {\begin{array}{*{20}c}    n & {\sum\limits_{{i = 1}}^{n} {x_{i} } } &  \cdots  & {\sum\limits_{{i = 1}}^{n} {x_{i}^{k} } }  \\    {\sum\limits_{{i = 1}}^{n} {x_{i} } } & {\sum\limits_{{i = 1}}^{n} {x_{i}^{2} } } &  \cdots  & {\sum\limits_{{i = 1}}^{n} {x_{i}^{{k + 1}} } }  \\     \vdots  &  \vdots  &  \ddots  &  \vdots   \\    {\sum\limits_{{i = 1}}^{n} {x_{i}^{k} } } & {\sum\limits_{{i = 1}}^{n} {x_{i}^{{k + 1}} } } &  \cdots  & {\sum\limits_{{i = 1}}^{n} {x_{i}^{{2k}} } }  \\   \end{array} } \right] = {\text{ }}\left[ {\begin{array}{*{20}c}    {a_{0} }  \\    {a_{1} }  \\     \vdots   \\    {a_{k} }  \\   \end{array} } \right] = \left[ {\begin{array}{*{20}c}    {\sum\limits_{{i = 1}}^{n} {y_{i} } }  \\    {\sum\limits_{{i = 1}}^{n} {x_{i} y_{i} } }  \\     \vdots   \\    {\sum\limits_{{i = 1}}^{n} {x_{k} y_{k} } }  \\   \end{array} } \right] $$

The matrix can be simplified as,

9 $$ \left[ {\begin{array}{*{20}c} 1 & {x_{1} } & \cdots & {x_{1}^{k} } \\ 1 & {x_{2} } & \cdots & {x_{2}^{k} } \\ \vdots & \vdots & \ddots & \vdots \\ 1 & {x_{n} } & \cdots & {x_{n}^{k} } \\ \end{array} } \right]\left[ \begin{aligned} a_{0} \hfill \\ a_{1} \hfill \\ \vdots \hfill \\ a_{k} \hfill \\ \end{aligned} \right] = \left[ \begin{aligned} y_{1} \hfill \\ y_{2} \hfill \\ \vdots \hfill \\ y_{n} \hfill \\ \end{aligned} \right] $$

Let

$$ x = \left[ {\begin{array}{*{20}c} 1 & {x_{1} } & \cdots & {x_{1}^{k} } \\ 1 & {x_{2} } & \cdots & {x_{2}^{k} } \\ \vdots & \vdots & \ddots & \vdots \\ 1 & {x_{n} } & \cdots & {x_{n}^{k} } \\ \end{array} } \right]\,\,A = \left[ \begin{aligned} a_{0} \hfill \\ a_{1} \hfill \\ \vdots \hfill \\ a_{k} \hfill \\ \end{aligned} \right]\,\,Y = \left[ \begin{aligned} y_{1} \hfill \\ y_{2} \hfill \\ \vdots \hfill \\ y_{n} \hfill \\ \end{aligned} \right] $$

So, the coefficient matrix *A* is obtained as *A *= *(X*^*T*^*X)*^−*1*^ *X*^*T*^*Y*, and at the same time, we can get the fitting curve.

### Segmental continuity rule

The plant root grows continuously which means that the derivative difference of a root changes slowly. Therefore, the derivative difference values of adjacent root segments are very close. And the endpoints derivatives of the adjacent root segments of the same root vary within a narrow range.

### Proximity rule

On the basis of the above rules, the endpoints of discrete root segments connect to that of nearest segments.

The detailed steps for root segments connection is as follow.

**Fig. 8:**
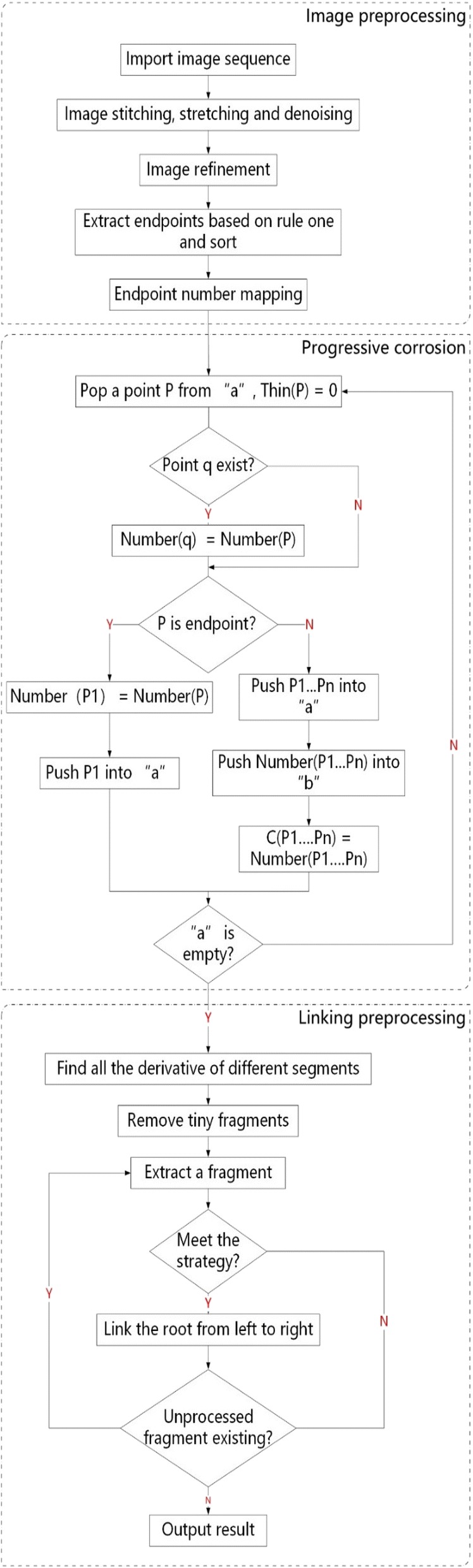
PCJ algorithm flowchart

- Step 1: Root segment characteristics extraction
  The derivative differences of two endpoints and the straight line slope of a root segment calculated according to the method of least squares are obtained as root segment characteristics for root segments matching.
- Step 2: Root segments matching
  The root segments match well as the same root if they meet the following conditions.
  - The difference of the straight line slopes of the two segments is less than a certain value *d1* and at the same time is more than a certain value *d2*.
  - The derivative difference values of adjacent endpoints of the two root segments for intended connection is not greater than a set value *d3*.
  - The loss value of the two segments calculated according to formula (7) is not more than a special value *d4*.
  - Based on the root growing trait of divergence, a root segment only connects to one near root segment but can be concatenated by more segments.
  The root segments matching method need 4 parameters which were adjusted manually for obtaining good results which optimization values are d1 = 4, d2 = 10, d3 = 50, d4 = 0.2.
- Step 3: Root segments connecting
  The adjacent endpoints of two root segments are linked together by using the quantic polynomial interpolation and minimum mean square error fitting method if they meet the above conditions.
  The tiny segments (not more than 10 pixels) are filled with black. These segments can be classified to two types: one is tiny root fragments, and the other one is noise caused by reflection light and impurities in the soil. The tiny fragments will be covered by the connection line created using the proposed PCJ algorithm and the noise will be removed as well. In addition, the reduction of plenty of tiny root segments can also speed up the computer processing.
  The whole flowchart of PCJ algorithm is as shown in Fig. 8.

### Root phenotypic parameters for analysis

Five root physiological parameters such as Root Number (RTN, obtained by calculating the number of end points in the repaired image), Root Length (RTL, acquired by calculating the total number of pixel points of each fitting line in the repaired image according to the L-ORB algorithm), Root Width (RTW, got by calculating the mean number of pixels perpendicular to the extension direction on each root in the binary image based on the L-ORB algorithm), Root Extension Length (REL, obtained by calculating the horizontal distance between the leftmost white pixel(s) and the rightmost pixel(s)) and Root Extension Angle (REA, calculated by measuring the angle between the two fitting lines of roots) are selected for root phenotyping analysis because they are important indexes of maize growing condition which can ensure the efficient absorption of moisture and nutrient uptake [34]. Before the measurement of the above five parameters, the relationship between the physical size and root image of a root is calibrated. Finally, the root phenotypic parameters are used to evaluate maize seed vigor to verify the validity of the method.

## Results

### Image processing results of PCJ algorithm

**Fig. 9:**
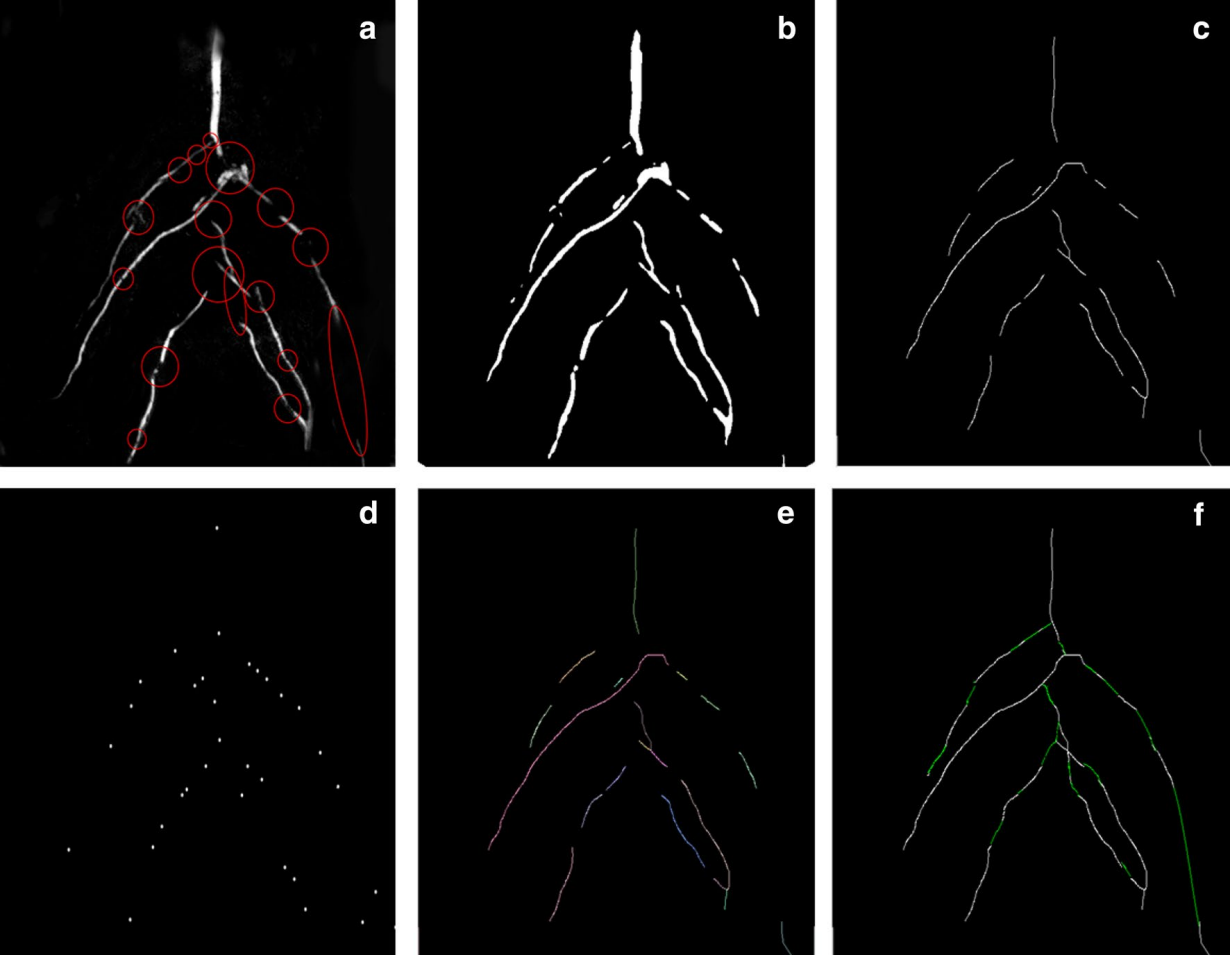
The result of the algorithm processing the picture of the maize root. **a** The image after denoising; **b** the image after binarization; **c** the image after thinning; **d** the end point of extracted maize root. Every endpoint is zoomed up by 5 times for the sake of observation; **e** The image processed by PCJ algorithm, and the points belonging to the same root are in the same color; **f** The completed image

In our experiments, as shown in Fig. 9a, there are some obvious gaps in the original maize root image after denoising processing, and the length and width of different root segments vary a lot. After binary processing, a clear boundary between root system and the soil is obtained and the width of different root segments can be calculated as shown in Fig. 9b.

Then the skeletal image of the root is got according to thinning algorithm, at this point the width of all root segments is one pixel in Fig. 9c. In Fig. 9d, all the endpoints of root segments are extracted as the beginning points of corrosion step. After progressive corrosion treatment, the different segments of the same root are marked as the same ID with the same color in Fig. 9e. Finally, the disconnected root segments are filled with green curves along the fitting curves for root recovering in Fig. 9f.

### Root phenotypic parameters of different vigor maize seeds

#### Static phenotypic parameters of maize seed

Root growing condition of a seed at the early stage is a highly effective way to evaluate seed vigor. In this paper, five root physiological parameters such as Root Number (RTN), Root Length (RTL), Root Width (RTW), Root Extension Length (REL) and Root Extension Angle (REA) are used as root phenotypic parameters for seed vigor indexes. As shown in Tables 3, 4 and 5, the experimental results of the root phenotypic parameters above calculated based on the refined root images using the presented PCJ algorithm show that there is remarkable correlation between maize vigor and root phenotypic parameters mentioned above. For the performance evaluation of the proposed PCJ algorithm, the maize seeds with 0, 3 and 7 aged days respectively were used for comparison test which results show that the error between the measured value and actual value is not more than 5%. Among them, the error of RTL is maximum because some of roots are blocked by the soil.

**Table 3:** Data record of the 7th day of the unaged root system

Physiolofical index	Measured value	Actual value	Error (%)
RTN (NUM)	12 ± 1	12 ± 1	0
RTL (CM)	82.4 ± 0.5	85.69 ± 0.5	4
RTW (MM)	1.53 ± 0.1	1.58 ± 0.1	3.2
REL (CM)	8.22 ± 0.3	8.24 ± 0.3	2.4
REA (DEGREE)	32.47 ± 2	32.5 ± 2	1

**Table 4:** Data of the 7th day of aging 3 days of seed

Physiolofical index	Measured value	Actual value	Error (%)
RTN (NUM)	10 ± 1	10 ± 1	0
RTL (CM)	76.8 ± 0.5	80.2 ± 0.5	4.5
RTW (MM)	1.49 ± 0.1	1.54 ± 0.1	3.6
REL (CM)	7.74 ± 0.3	7.91 ± 0.3	2.2
REA (DEGREE)	45.63 ± 2	46.3 ± 2	1.5

**Table 5:** Day 7 data records of aging 7 days of seeds

Physiolofical index	Measured value	Actual value	Error (%)
RTN (NUM)	5 ± 1	5 ± 1	0
RTL (CM)	45.6 ± 0.5	47.5 ± 0.5	4.2
RTW (MM)	1.45 ± 0.1	1.5 ± 0.1	3.4
REL (CM)	6.08 ± 0.3	6.23 ± 0.3	2.5
REA (DEGREE)	42.15 ± 2	42.9 ± 2	1.8

The data in the table retains 2 decimal places. The true value is the measured value after washing the soil

**Fig. 10:**
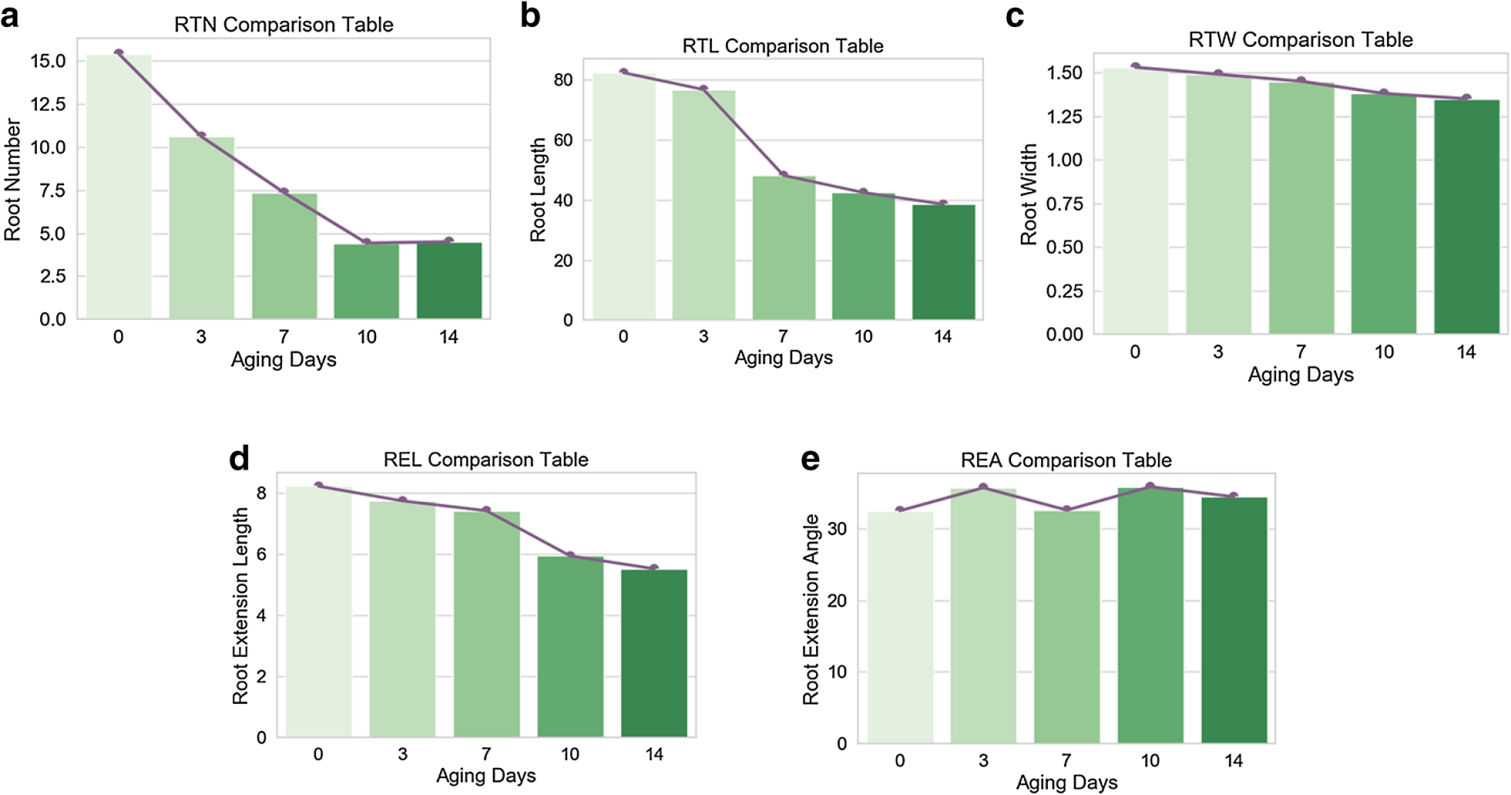
Comparison of parameters of roots of different vigorous maize seeds after 7 days of growth. **a** The comparison table of aging days and root numbers on the 7th day of maize root growth. **b** The comparison table of aging days and root length on the 7th day of maize root growth. **c** The comparison table of aging days and root width on the 7th day of maize root growth. **d** The comparison table of aging days and root extension length on the 7th day of maize root growth. **e** The comparison table of aging days and root extension angle on the 7th day of maize root growth

The experiments indicate that, in Fig. 10, Root Number (RTN), Root Length (RTL), Root Width (RTW) and Root Extension Length (REL) of unaged and 14-day-aged maize seeds are decreased from 15.40, 82.40 mm, 1.53 mm and 82.20 mm to 4.58, 38.6 mm, 1.35 mm and 55.20 mm, respectively. After analyzing the data, it can be found that there is an evident negative linear between seed vigor and RTN, RTL, RTW, REL, but there is uncorrelation between REA and maize seed vigor.

The regression relationships between aging day and root growth parameters are as follows:

10 $$ RTN \, = \, 0.72d^{2} - \, 7.118d \, + \, 21.888 $$

11 $$ RTW = \, 0.0033d^{3} - \, 0.0307d^{2} + \, 0.036d \, + \, 1.52 $$

12 $$ RTL = \, 2.0667d^{3} - \, 16.721d^{2} + \, 25.312d \, + \, 72.7 $$

13 $$ RTET = 0.075d^{3} - 0.7493d^{2} + 1.4957d \, + 7.348 $$

*d* is the aging day.

By comparison, it shows that the relation curves between RTL, REL and maize seed aging degree are very similar to that of seed vigor and deterioration [35].

### Dynamic phenotypic parameters of maize seed

To obtain maize seed vigor information quickly, the dynamic phenotypic parameters of maize seed root system during early stage were used to mine the information about seed vigor.

**Fig. 11:**
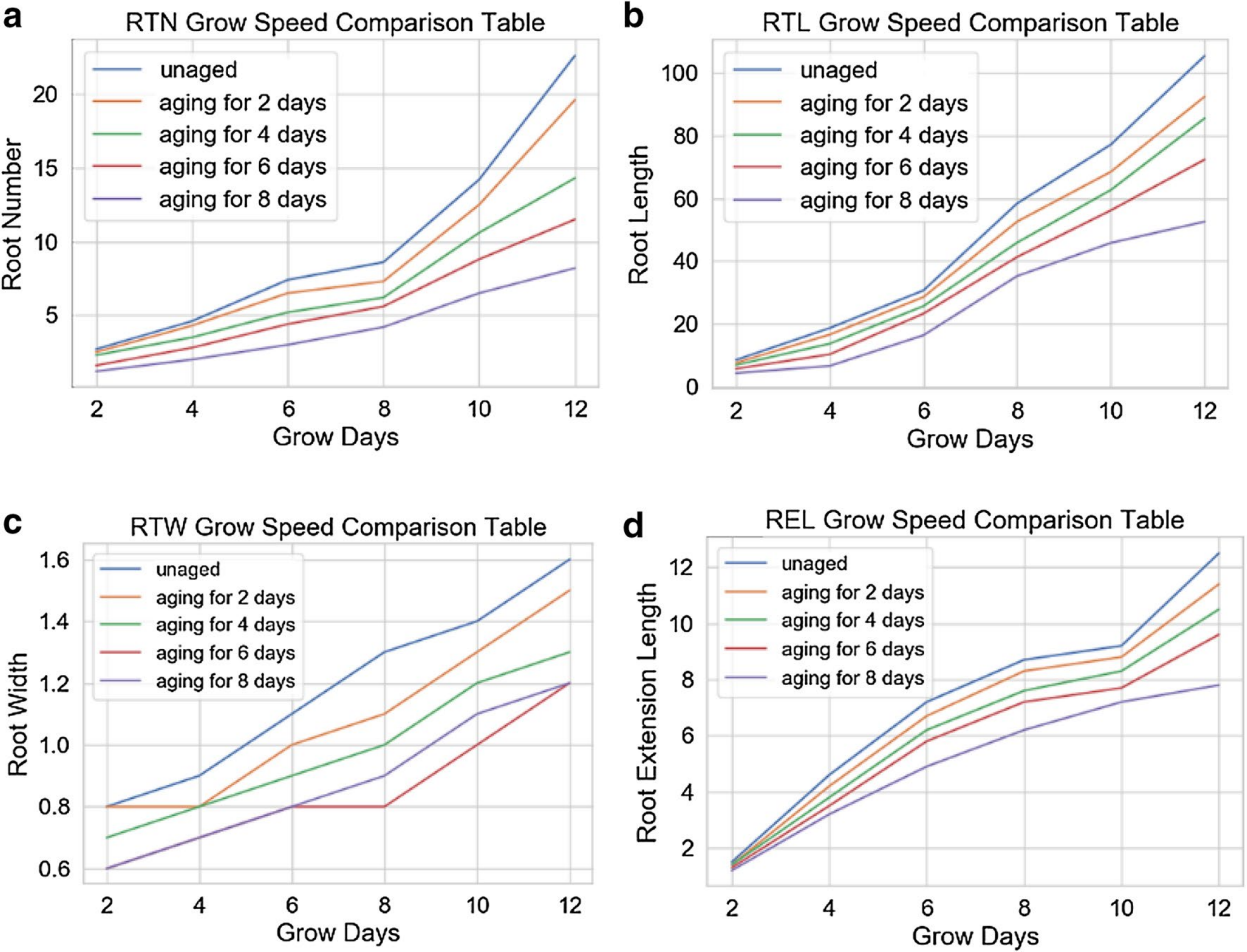
Comparison of the growth rate of root parameters of different vigorous maize seeds after 7 days of growth. **a** The comparison table of grow days and root number of different vigor maize seeds. **b** The comparison table of grow days and root length of different vigor maize seeds. **c** The comparison table of grow days and root width of different vigor maize seeds. **d** The comparison table of grow days and root extension length of different vigor maize seeds

As shown in Fig. 11, the differences between RTN, RTL, RTW, REL and ageing degree are even more remarkable, moreover, the gaps of RTN, RTL and REL are increasing during 2 to 12 growth days from 1.5, 4.2, 0.2, 0.3 to 14.4, 52.9, 0.4, 4.7 respectively. The average growing speed of RTN, RTL, RTW and REL change from 1.68 per day, 8.80 mm/d, 0.06 mm/d, 9.0 mm/d to 0.70 per day, 4.3 mm/d, 0.05 mm/d and 5.70 mm/d respectively. Based on the analysis, it can be found that there is a remarkable correlation between the above four dynamic phenotypic parameters and maize seed aged day.

In summary, the static as well as dynamic maize root phenotyping parameters can be used to evaluate the vigor of seeds.

## Discussion

In order to obtain more root image information directly and rapidly as well as acquire the complete root image from the root segments blocked by the soil, the paper developed an ARPC device which can coax root system growing along the inner wall of the container to expose more parts due to narrow growing annulus space and capillary effect on the boundary between the soil and the container. Moreover, the PCJ algorithm was proposed to recover gaps from disconnected root segments for reconstructing a complete root system architecture.

It should be noted that the toroidal surfaces of the container need be covered by black items to avoid sunlight because the root system will grow into the soil due to root negative phototropism trait which will result in root information reduction in the image. Besides, polarizers and pure black soil were used to overcome the reflection from the transparent container and the disturbance from the soil respectively during image sampling. For obtaining high quality image, the professional SLR camera was used in macro mode in a dark environment indoors with artificial light and the parameters such as polarized angle, aperture, ISO and shutter speed need to be adjusted according to ambient luminous intensity.

For improving the processing speed of the algorithm in computer, the short root segments, not more than 30 pixels in our experiment, can be neglected and covered by the calculated joining root.

However, the proposed ARPC device and PCJ algorithm are focusing on the root phenotyping detection of the early growing stage of fibrous root plant owing to the limitation of the narrow growing annulus space and the increasing proportion of root blocked by the soil, whereas CT X-ray can be applied to acquire the complete image of plant root in any growing stage accurately with huge cost.

## Conclusion

In order to realize maize root fast non-invasive phenotyping detection, a novel ARPC device was developed to push each root growing along the inner wall of the container to acquire more root information. Moreover, the PCJ algorithm was proposed to recover various gaps among root segments effectively to generate complete root system images. Finally, five physiological parameters such as RTN, RTL, RTW, REL and REA obtained from the recovered root image by using the presented ARPC device combined with PCJ algorithm were applied to evaluate maize seed vigor which results show that there is an evident negative linear between seed vigor and RTN, RTL, RTW, REL. In addition, the correlation between the above four root phenotypic parameters and maize seed aged day is also remarkable. The proposed ARPC device combined with PCJ algorithm has vast application prospect in high throughput root phenotyping area.

## Supplementary information

**Additional file 1.** 16 original root images and the corresponding 16 processed images using the presented algorithm in the paper.

**Additional file 2.** The key codes of the Progressive Corrosion algorithm.

## Data Availability

Original procedure and data for root system architecture and analysis is available on request.

## Abbreviations

RTN: the number of roots
RTL: root length: the total length of all roots
RTW: root width: the average width of each root
REL: root extension length, the horizontal length of the root system
REA: root extension angle, the average angle of each root bend
PCJ: Progressive Corrosion Joining
ARPC: Annular Root Phenotyping Container
