# Supplementary figures and images for: Root phenotypic detection of different vigorous maize seeds based on Progressive Corrosion Joining algorithm of image
Source: Plant Methods. 2019 Nov 18;15:137. doi: 10.1186/s13007-019-0518-5 (PMC6859636; doi:10.1186/s13007-019-0518-5)

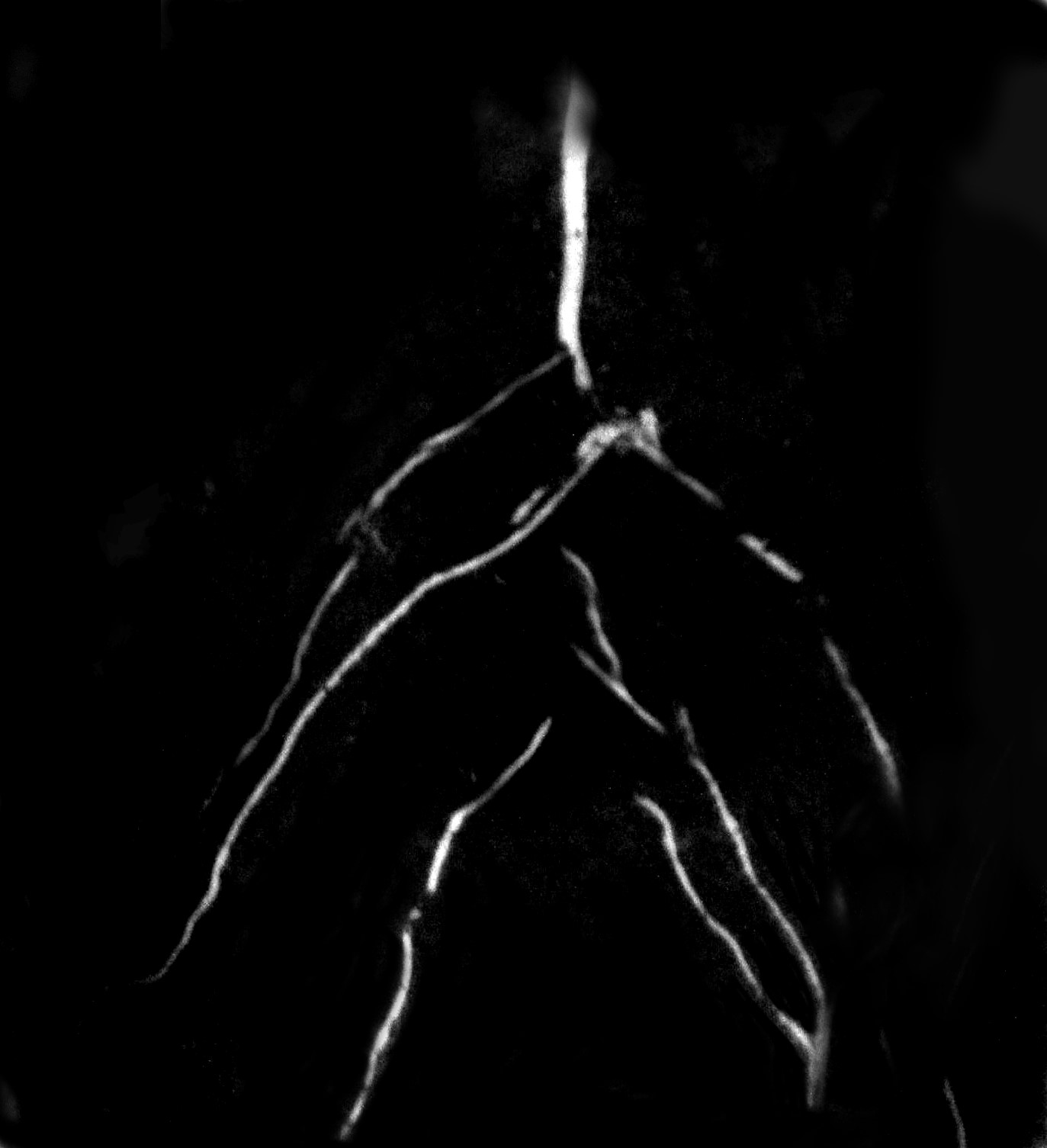

Supplement: Supplementary file 1 — Additional file 1. 16 original root images and the corresponding 16 processed images using the presented algorithm in the paper. [file 13007_2019_518_MOESM1_ESM.zip › test_1.1.jpg]

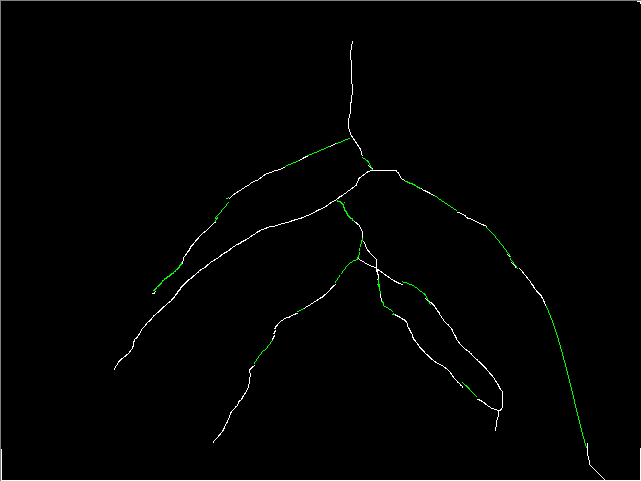

Supplement: Supplementary file 1 — Additional file 1. 16 original root images and the corresponding 16 processed images using the presented algorithm in the paper. [file 13007_2019_518_MOESM1_ESM.zip › test_1.2.png]

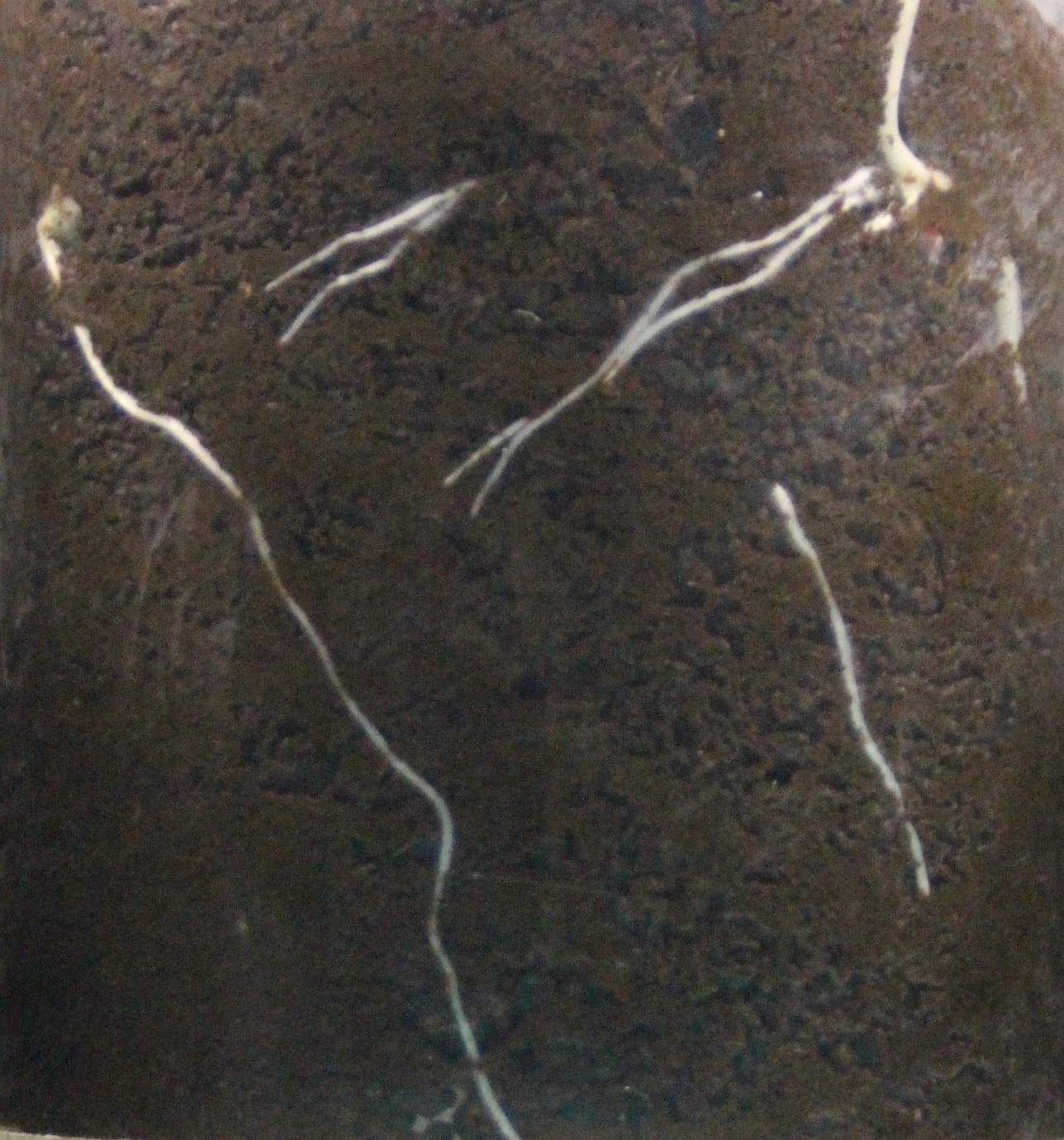

Supplement: Supplementary file 1 — Additional file 1. 16 original root images and the corresponding 16 processed images using the presented algorithm in the paper. [file 13007_2019_518_MOESM1_ESM.zip › test_10.1.JPG]

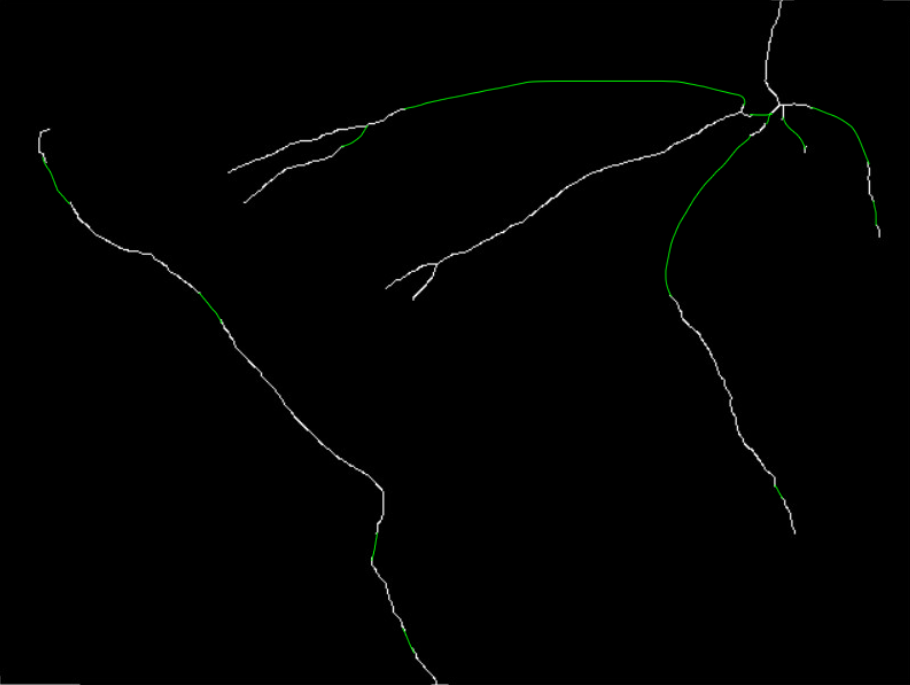

Supplement: Supplementary file 1 — Additional file 1. 16 original root images and the corresponding 16 processed images using the presented algorithm in the paper. [file 13007_2019_518_MOESM1_ESM.zip › test_10.2.png]

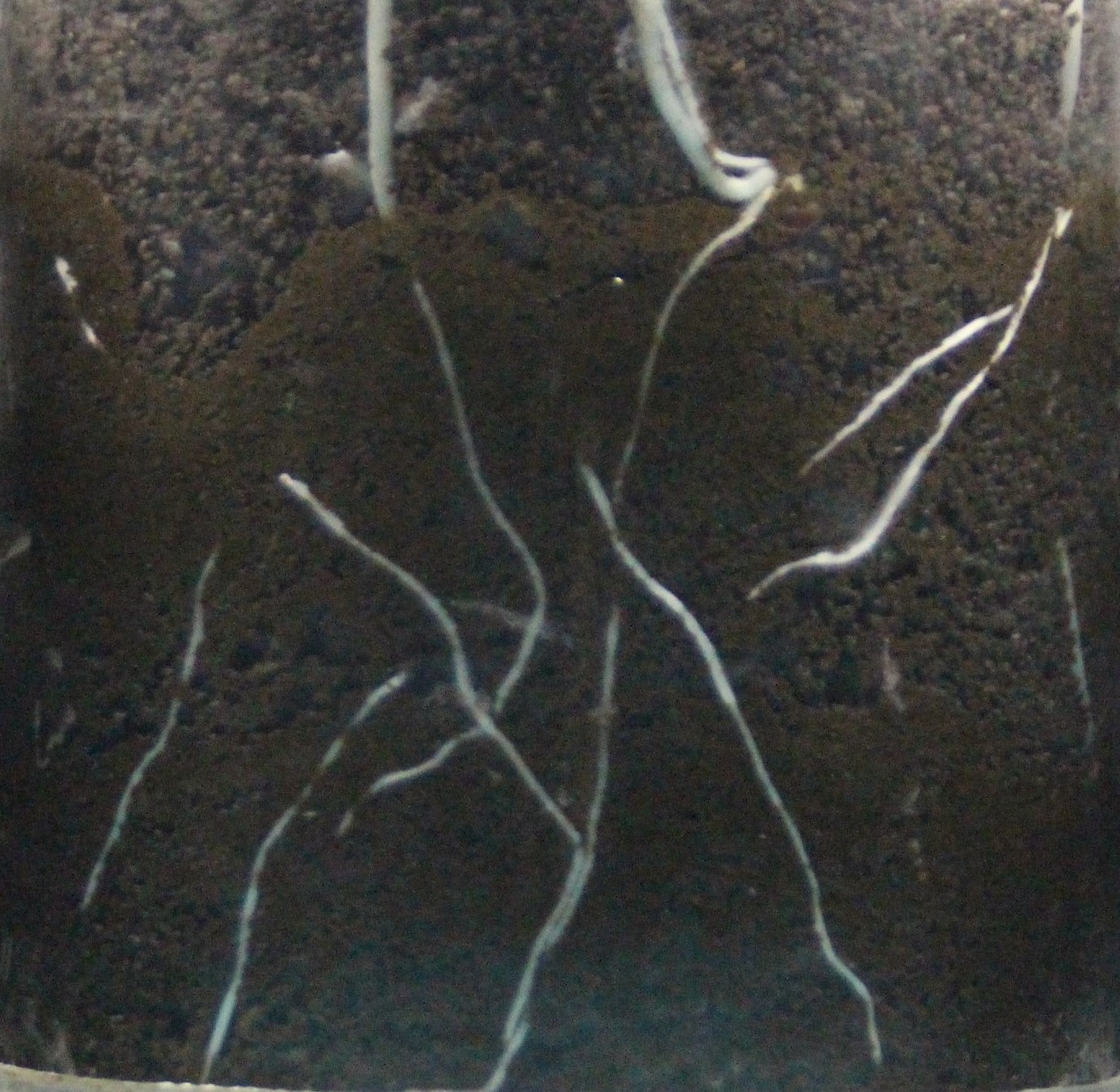

Supplement: Supplementary file 1 — Additional file 1. 16 original root images and the corresponding 16 processed images using the presented algorithm in the paper. [file 13007_2019_518_MOESM1_ESM.zip › test_11.1.JPG]

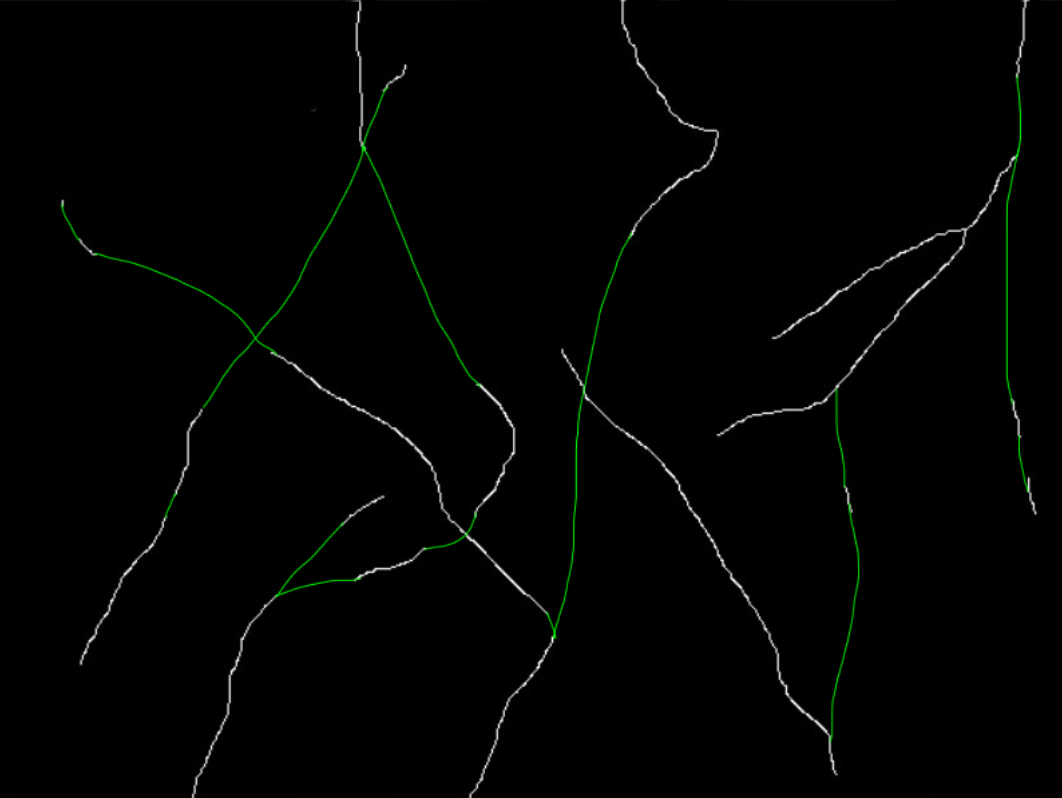

Supplement: Supplementary file 1 — Additional file 1. 16 original root images and the corresponding 16 processed images using the presented algorithm in the paper. [file 13007_2019_518_MOESM1_ESM.zip › test_11.2.png]

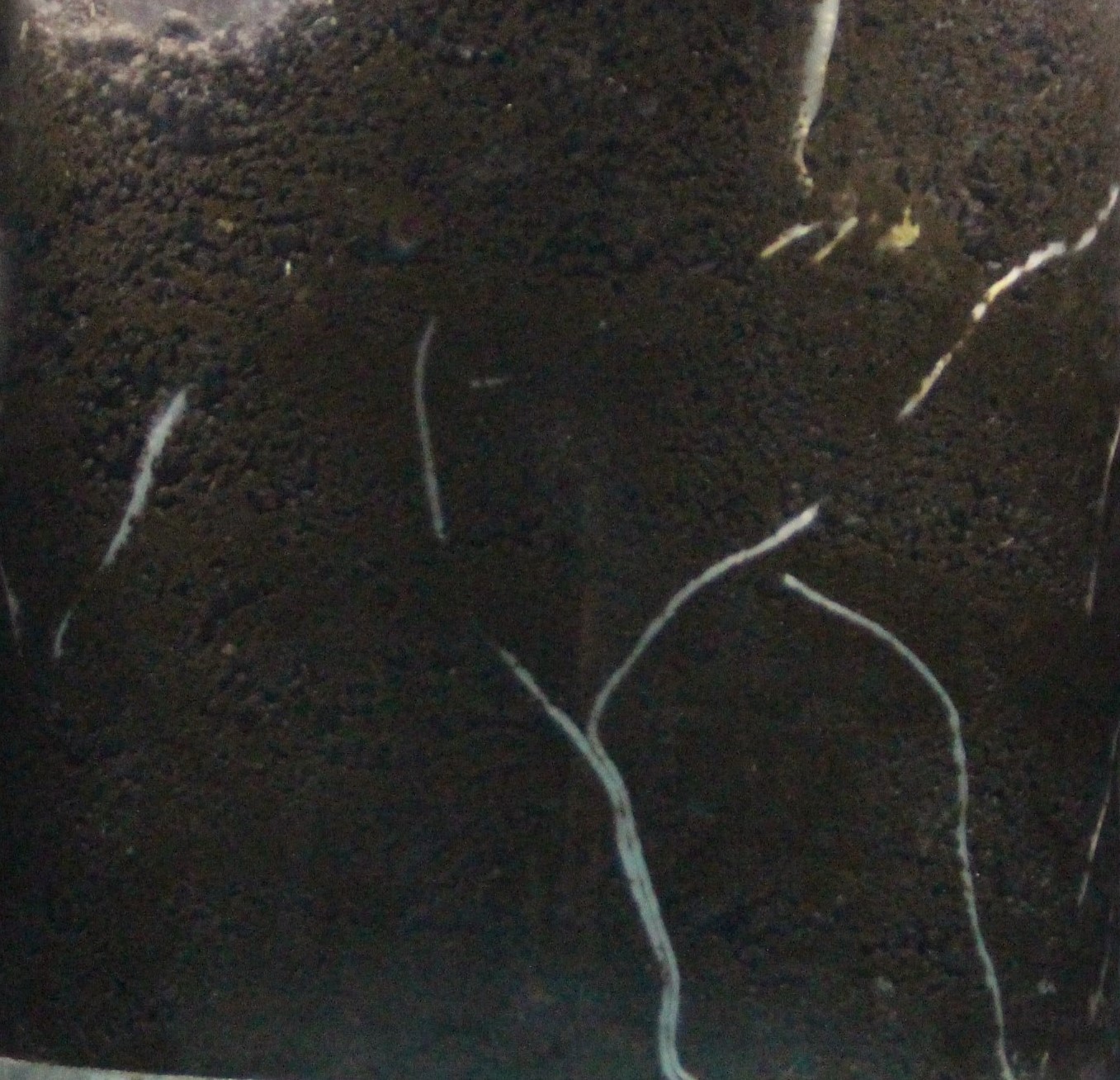

Supplement: Supplementary file 1 — Additional file 1. 16 original root images and the corresponding 16 processed images using the presented algorithm in the paper. [file 13007_2019_518_MOESM1_ESM.zip › test_12.1.JPG]

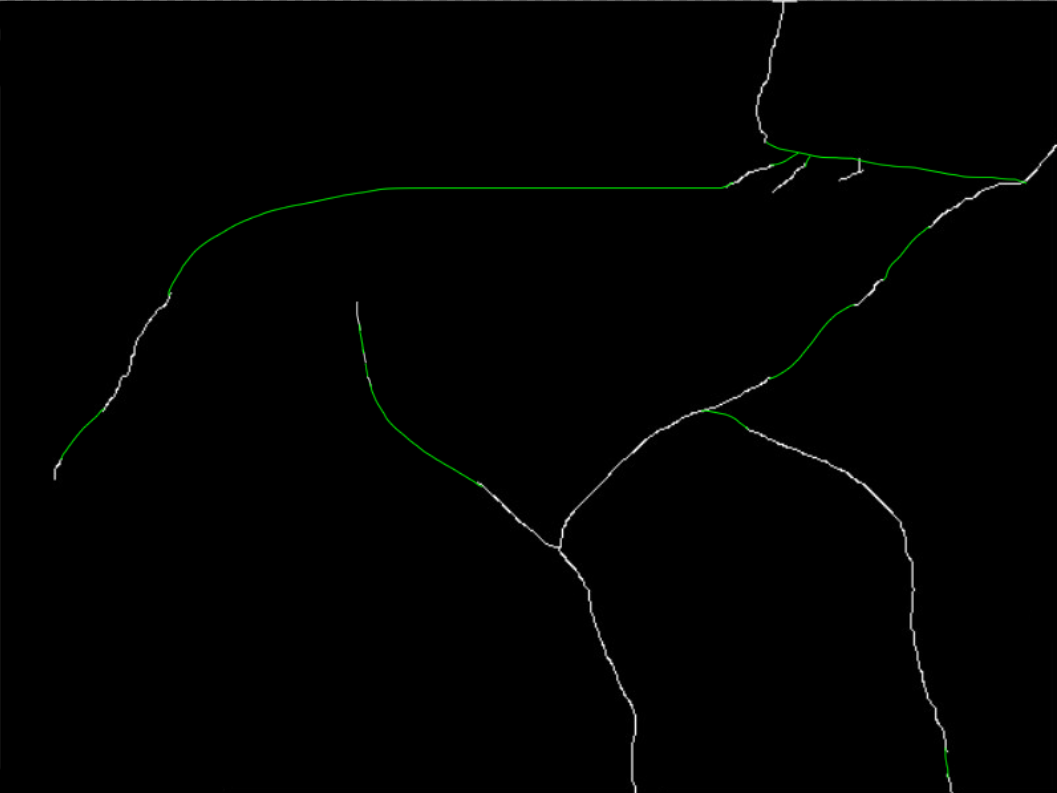

Supplement: Supplementary file 1 — Additional file 1. 16 original root images and the corresponding 16 processed images using the presented algorithm in the paper. [file 13007_2019_518_MOESM1_ESM.zip › test_12.2.png]

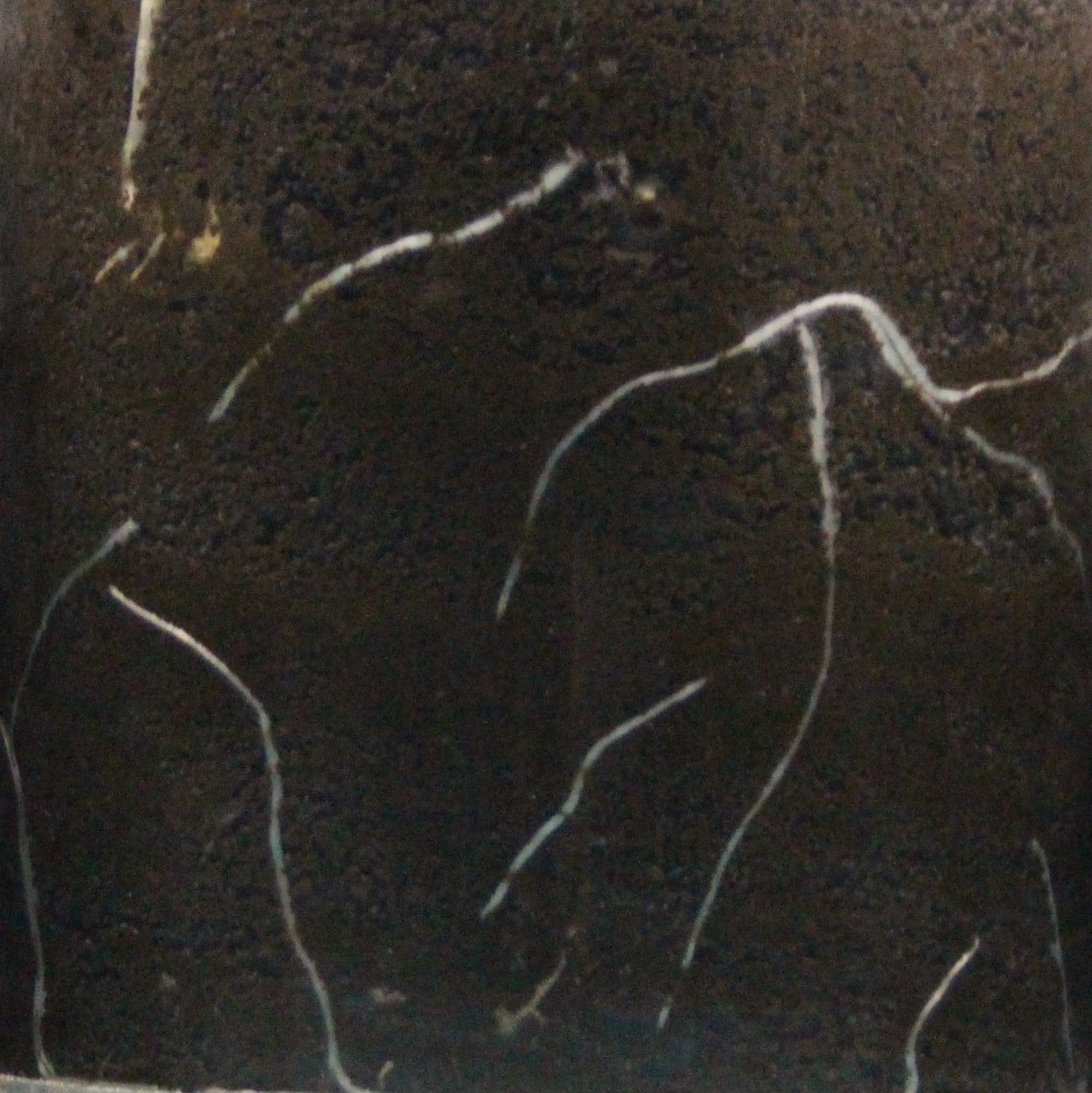

Supplement: Supplementary file 1 — Additional file 1. 16 original root images and the corresponding 16 processed images using the presented algorithm in the paper. [file 13007_2019_518_MOESM1_ESM.zip › test_13.1.JPG]

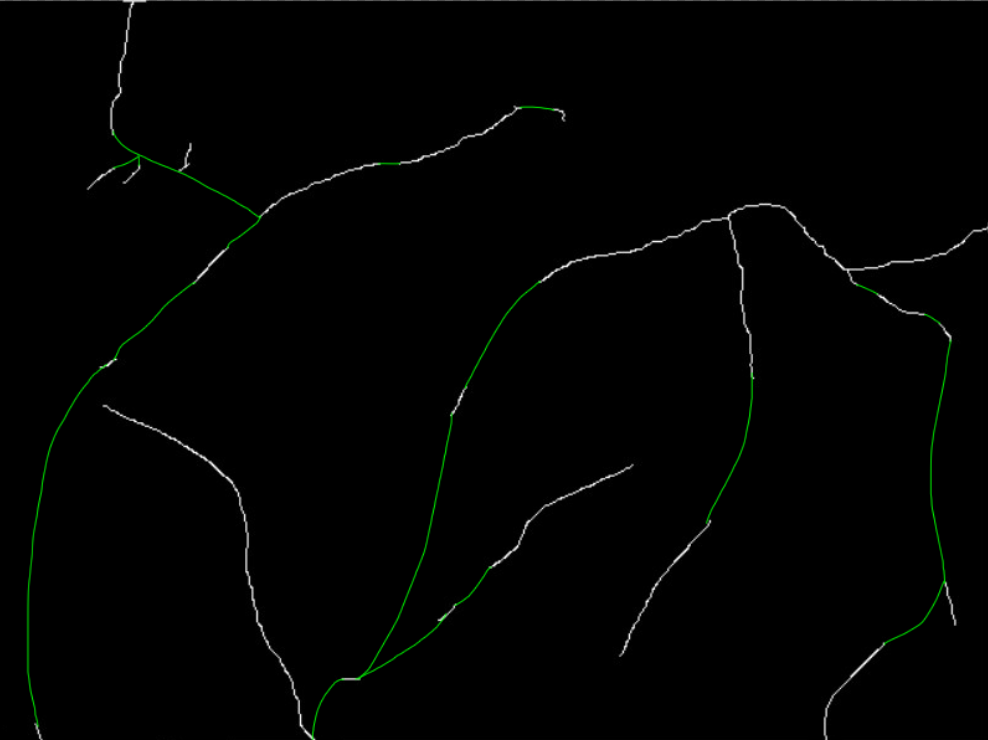

Supplement: Supplementary file 1 — Additional file 1. 16 original root images and the corresponding 16 processed images using the presented algorithm in the paper. [file 13007_2019_518_MOESM1_ESM.zip › test_13.2.png]

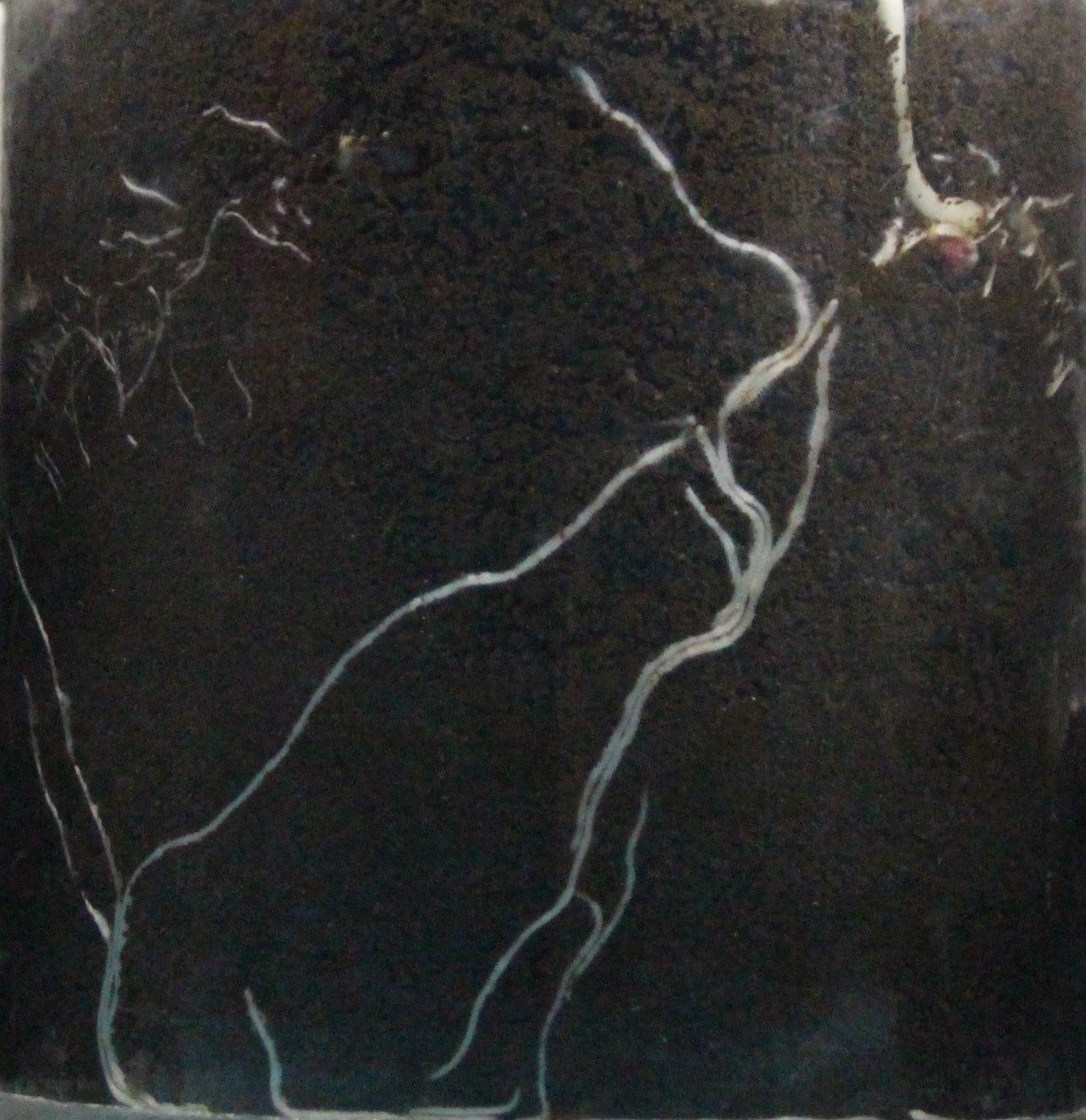

Supplement: Supplementary file 1 — Additional file 1. 16 original root images and the corresponding 16 processed images using the presented algorithm in the paper. [file 13007_2019_518_MOESM1_ESM.zip › test_14.1.JPG]

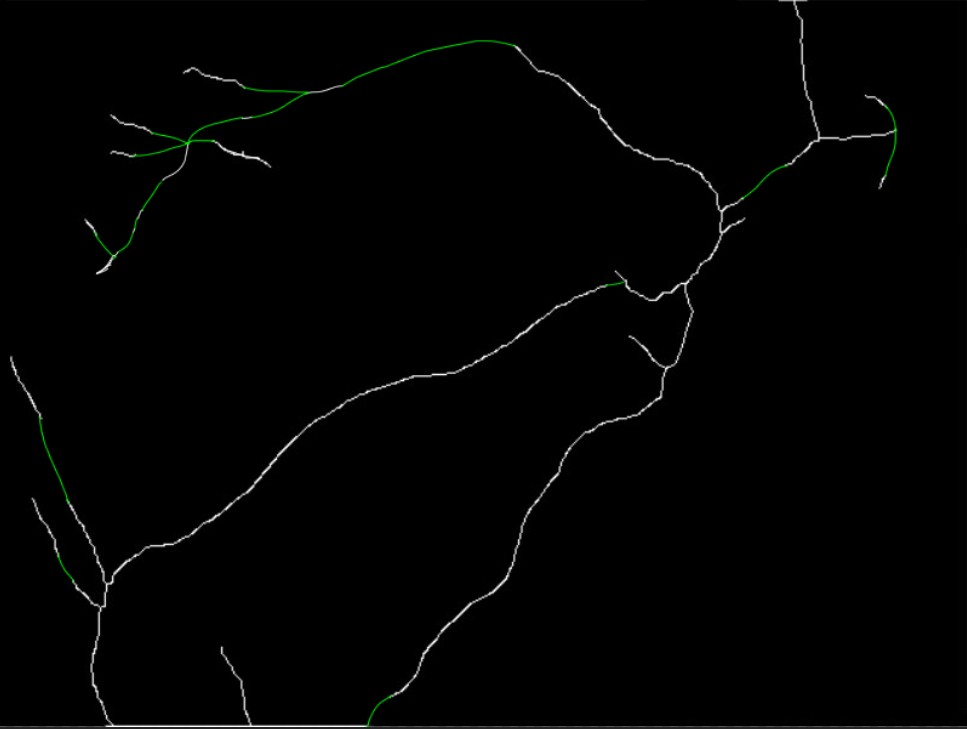

Supplement: Supplementary file 1 — Additional file 1. 16 original root images and the corresponding 16 processed images using the presented algorithm in the paper. [file 13007_2019_518_MOESM1_ESM.zip › test_14.2.png]

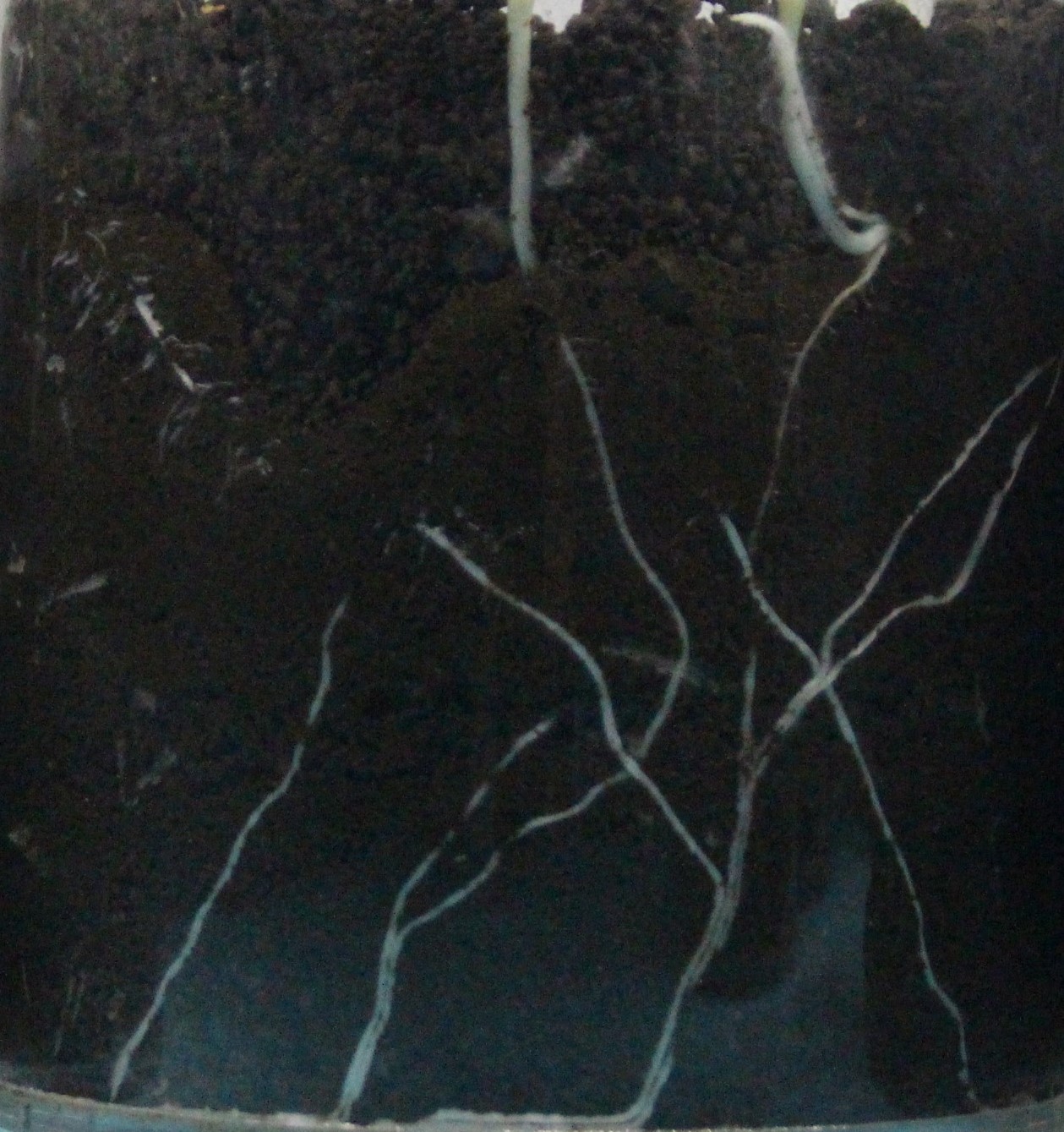

Supplement: Supplementary file 1 — Additional file 1. 16 original root images and the corresponding 16 processed images using the presented algorithm in the paper. [file 13007_2019_518_MOESM1_ESM.zip › test_15.1.JPG]

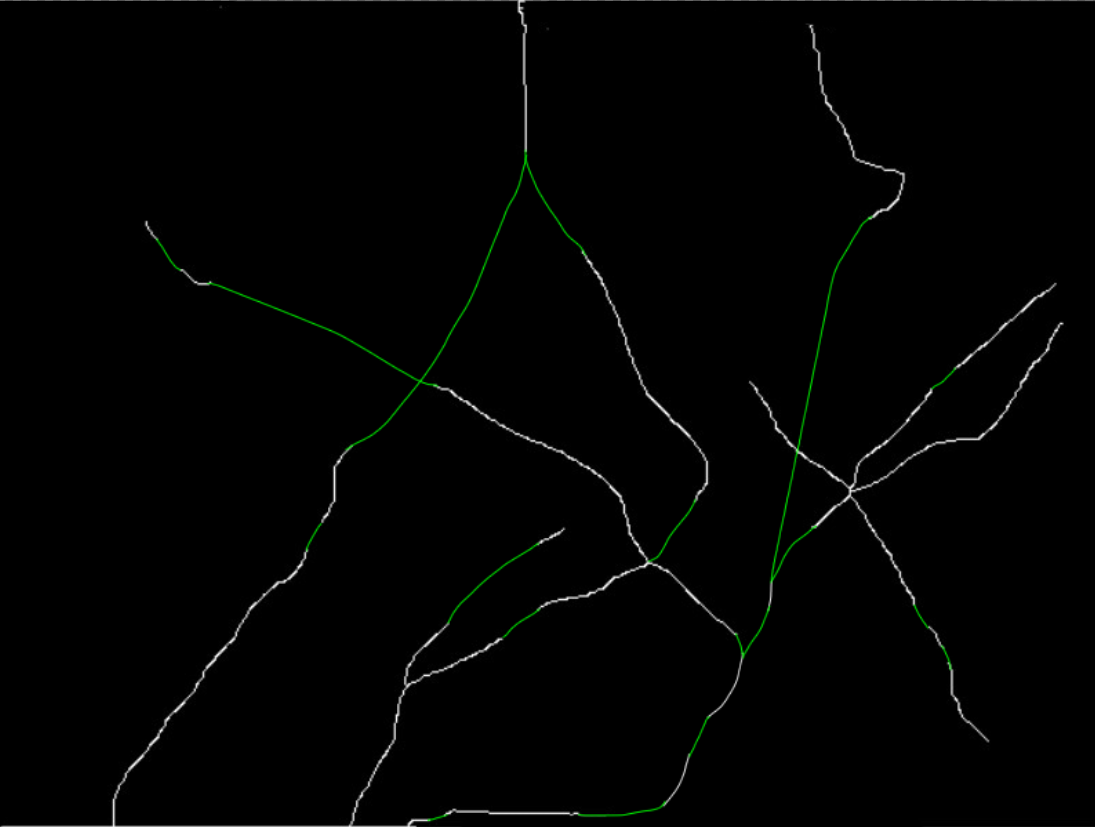

Supplement: Supplementary file 1 — Additional file 1. 16 original root images and the corresponding 16 processed images using the presented algorithm in the paper. [file 13007_2019_518_MOESM1_ESM.zip › test_15.2.png]

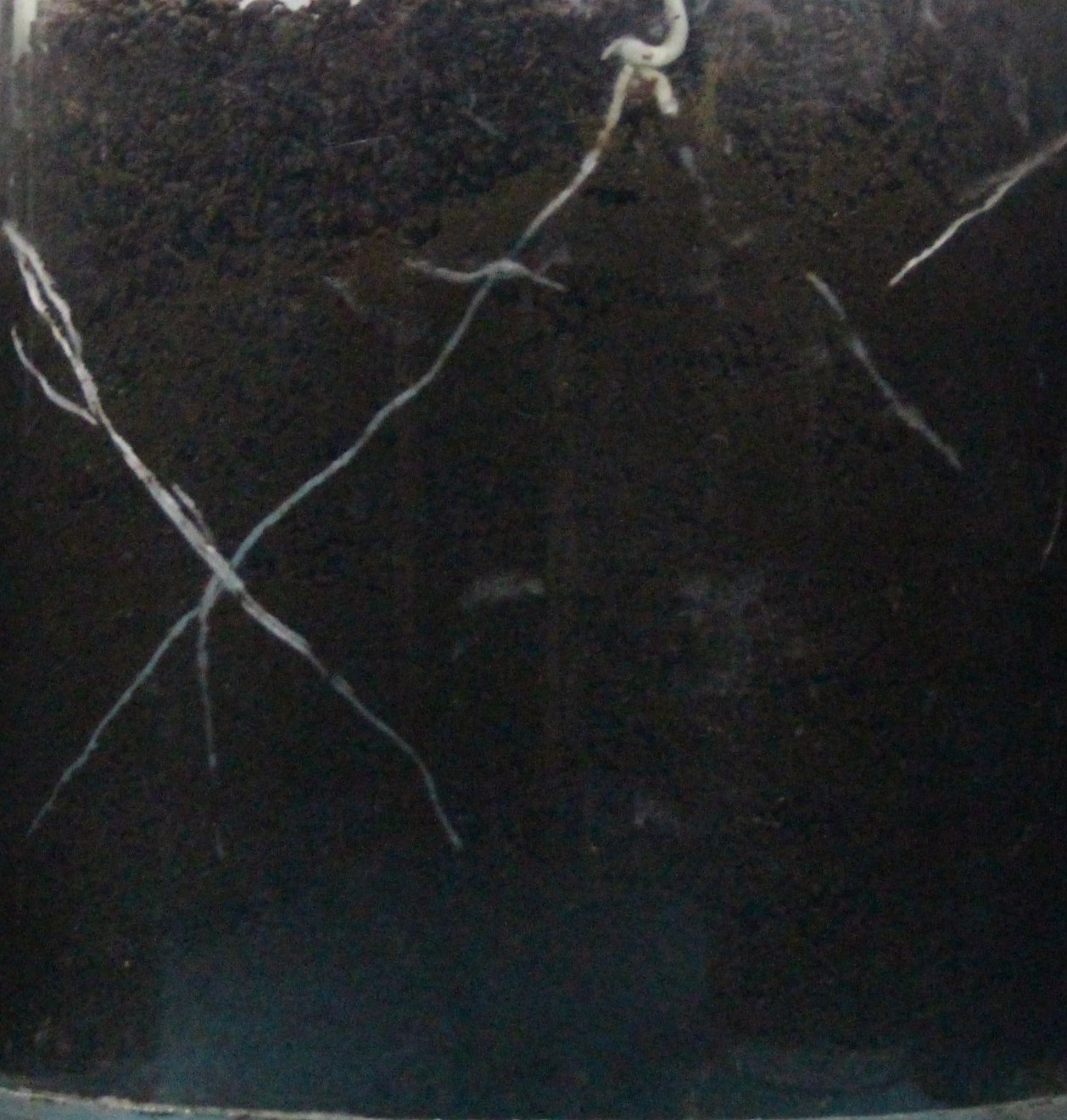

Supplement: Supplementary file 1 — Additional file 1. 16 original root images and the corresponding 16 processed images using the presented algorithm in the paper. [file 13007_2019_518_MOESM1_ESM.zip › test_16.1.JPG]

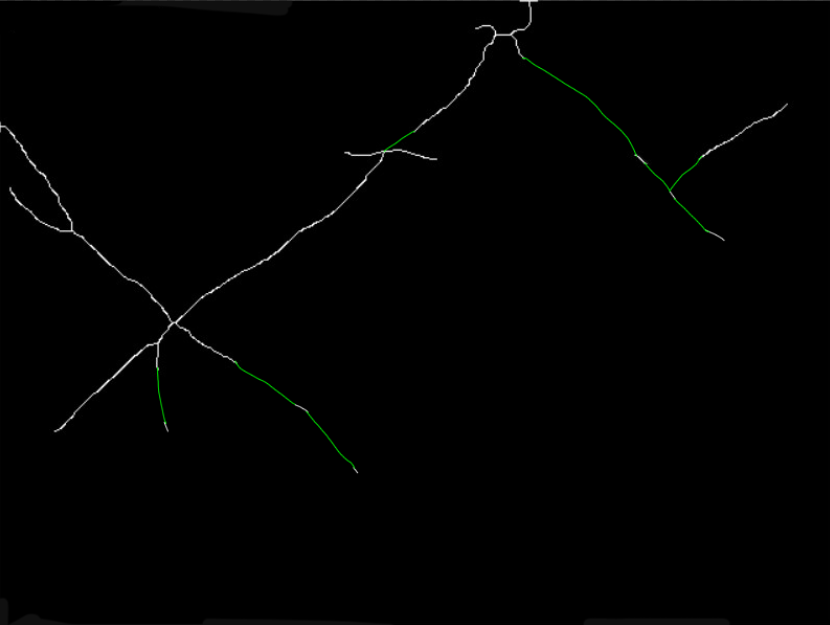

Supplement: Supplementary file 1 — Additional file 1. 16 original root images and the corresponding 16 processed images using the presented algorithm in the paper. [file 13007_2019_518_MOESM1_ESM.zip › test_16.2.png]

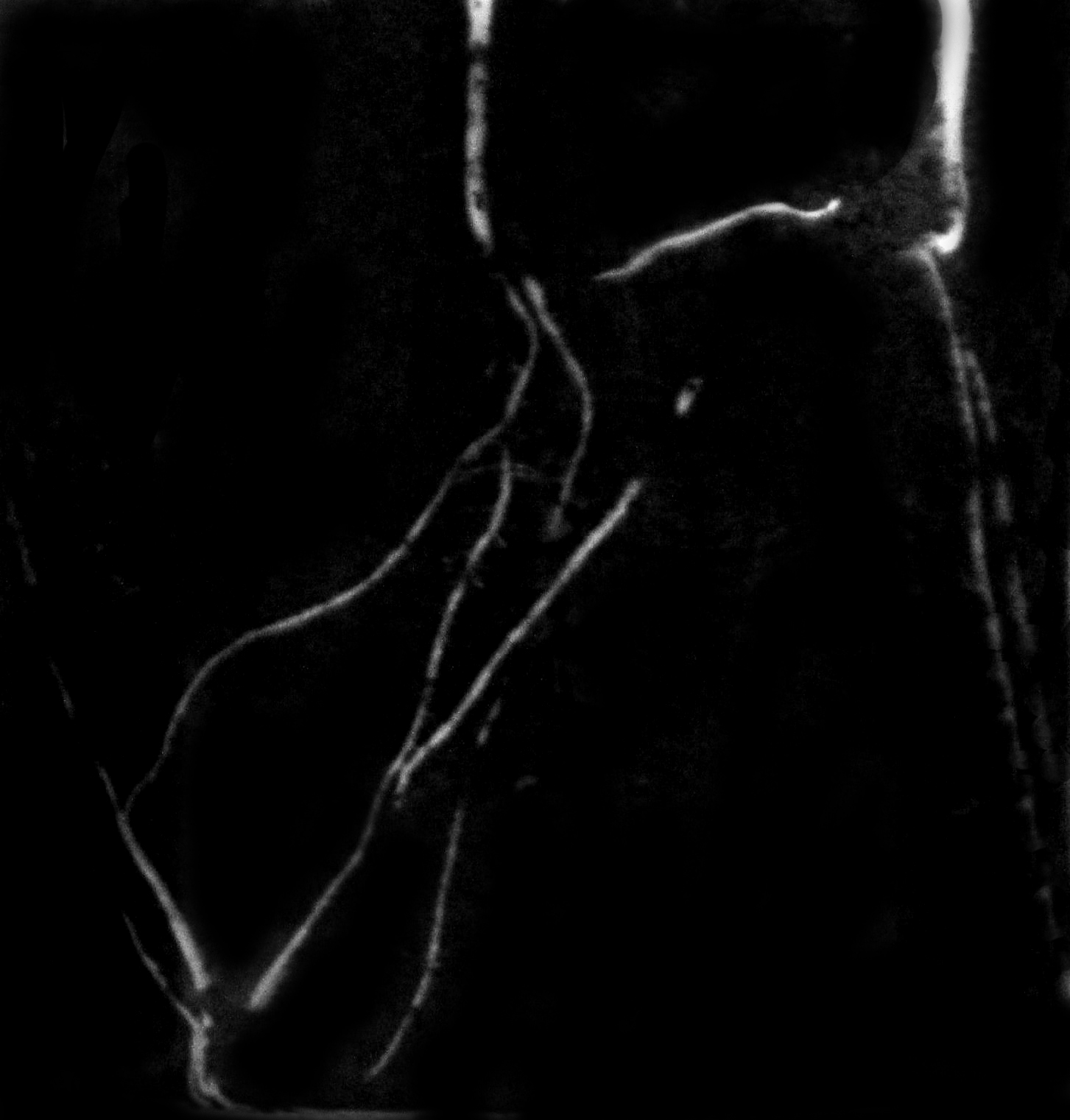

Supplement: Supplementary file 1 — Additional file 1. 16 original root images and the corresponding 16 processed images using the presented algorithm in the paper. [file 13007_2019_518_MOESM1_ESM.zip › test_2.1.jpg]

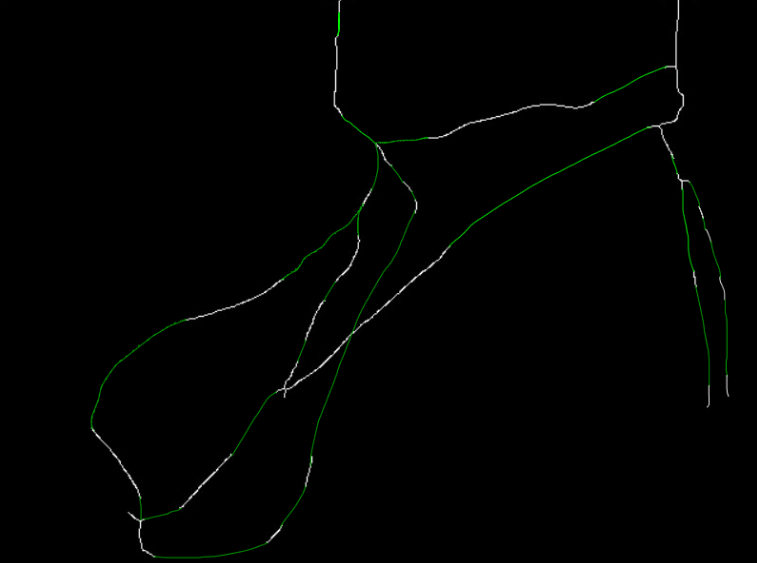

Supplement: Supplementary file 1 — Additional file 1. 16 original root images and the corresponding 16 processed images using the presented algorithm in the paper. [file 13007_2019_518_MOESM1_ESM.zip › test_2.2.png]

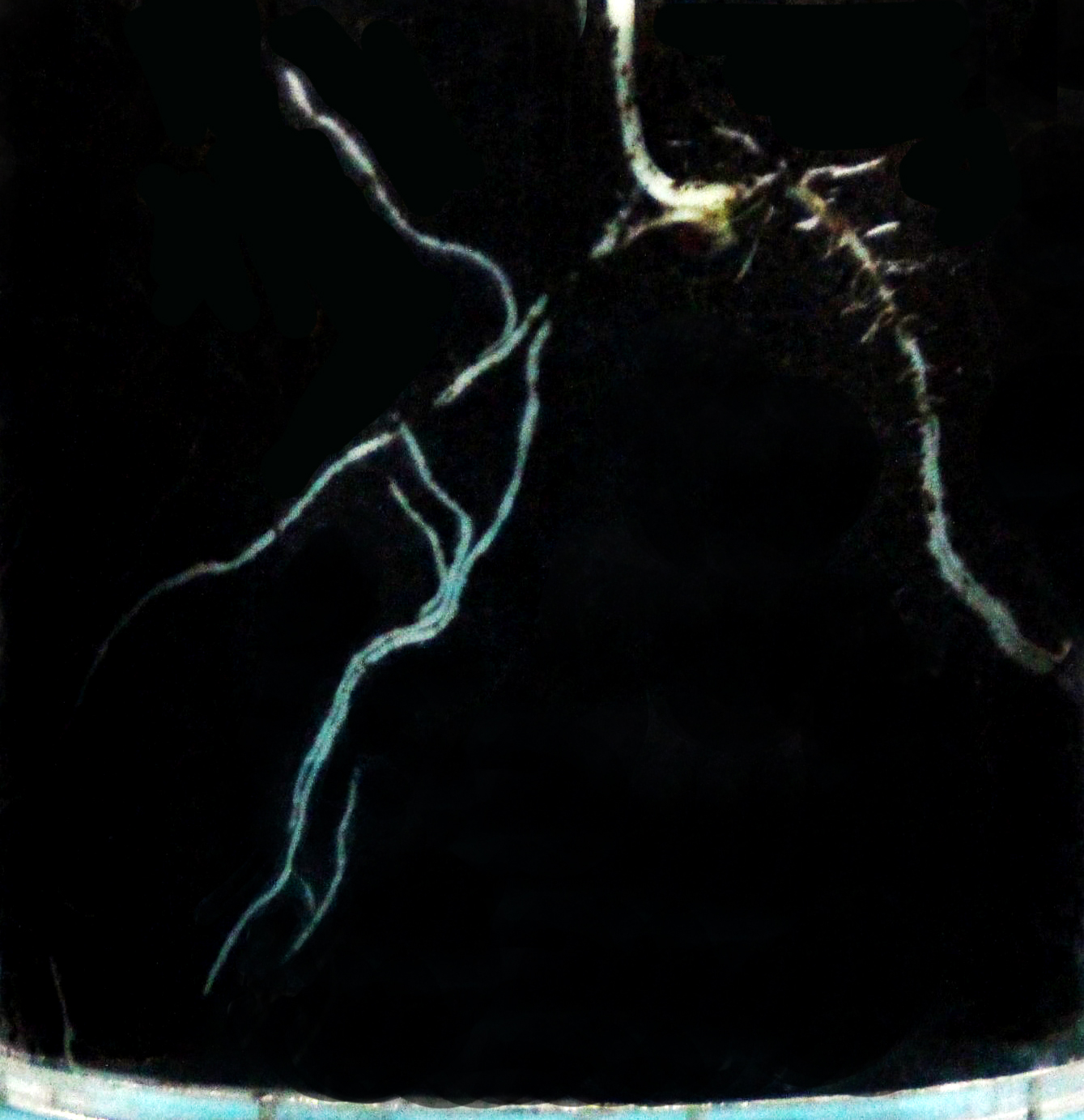

Supplement: Supplementary file 1 — Additional file 1. 16 original root images and the corresponding 16 processed images using the presented algorithm in the paper. [file 13007_2019_518_MOESM1_ESM.zip › test_3.1.jpg]

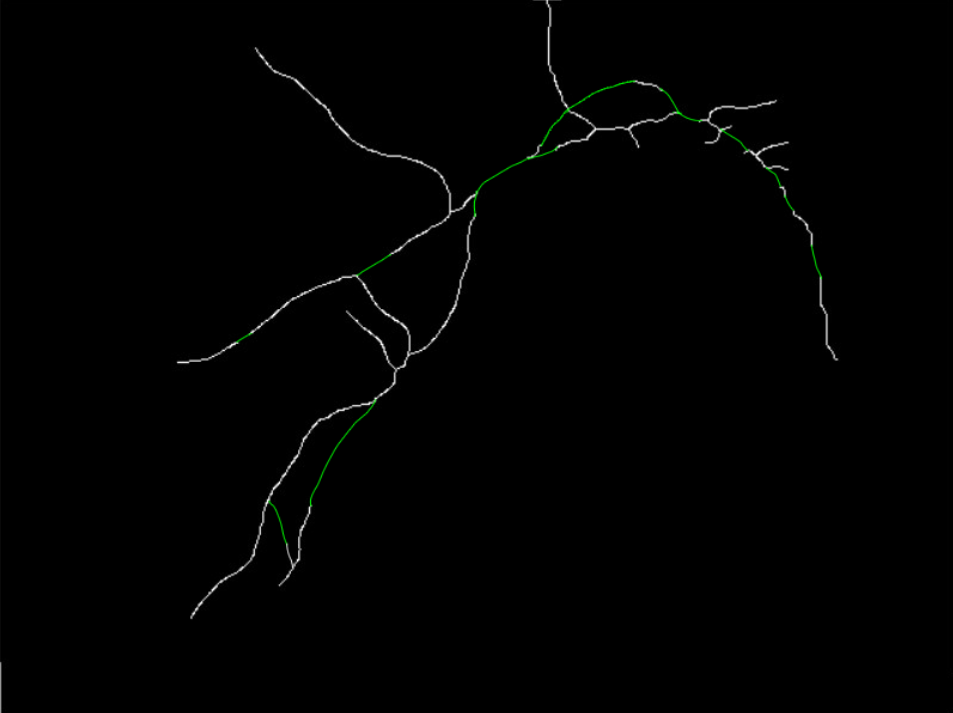

Supplement: Supplementary file 1 — Additional file 1. 16 original root images and the corresponding 16 processed images using the presented algorithm in the paper. [file 13007_2019_518_MOESM1_ESM.zip › test_3.2.png]

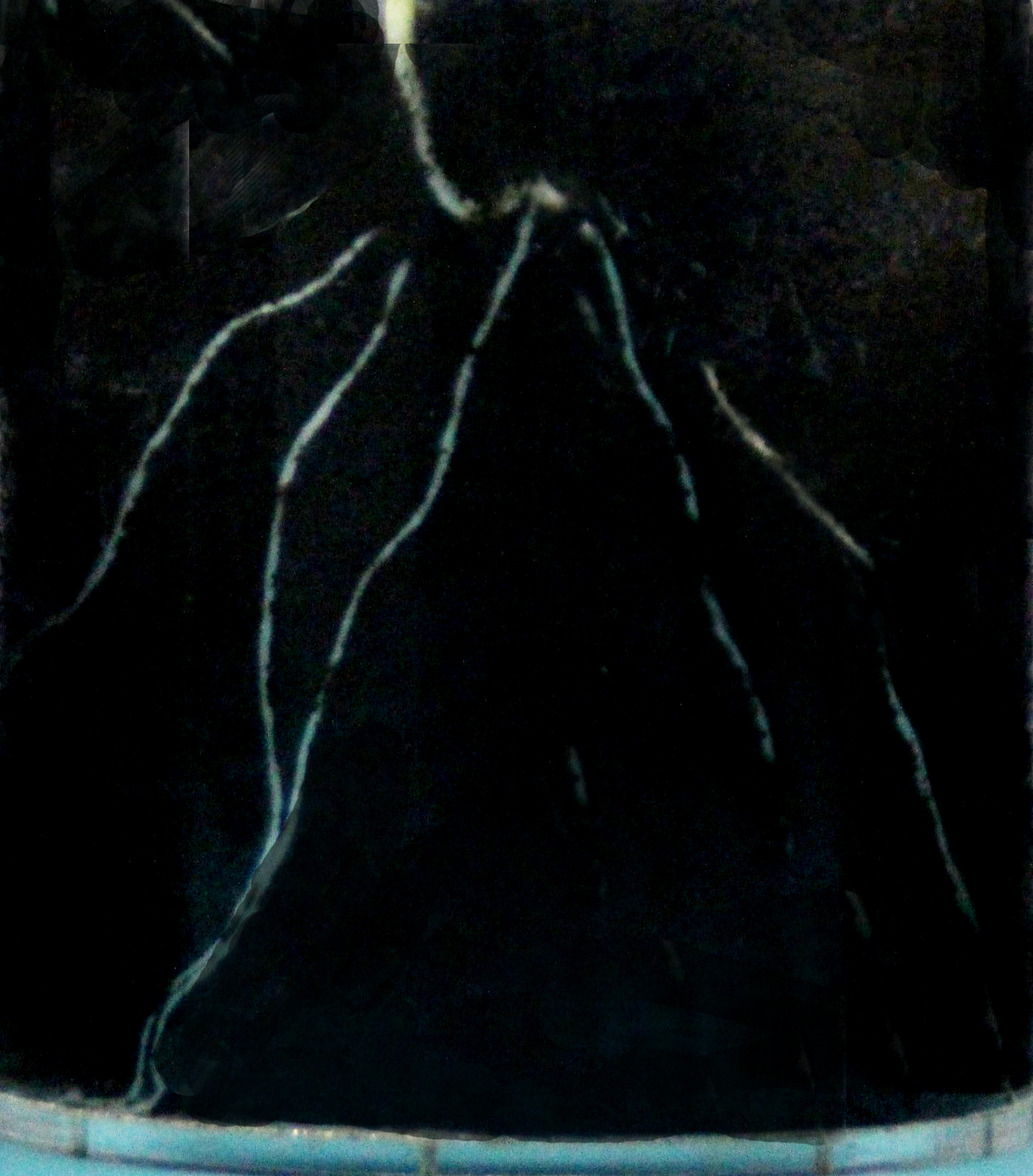

Supplement: Supplementary file 1 — Additional file 1. 16 original root images and the corresponding 16 processed images using the presented algorithm in the paper. [file 13007_2019_518_MOESM1_ESM.zip › test_4.1.jpg]

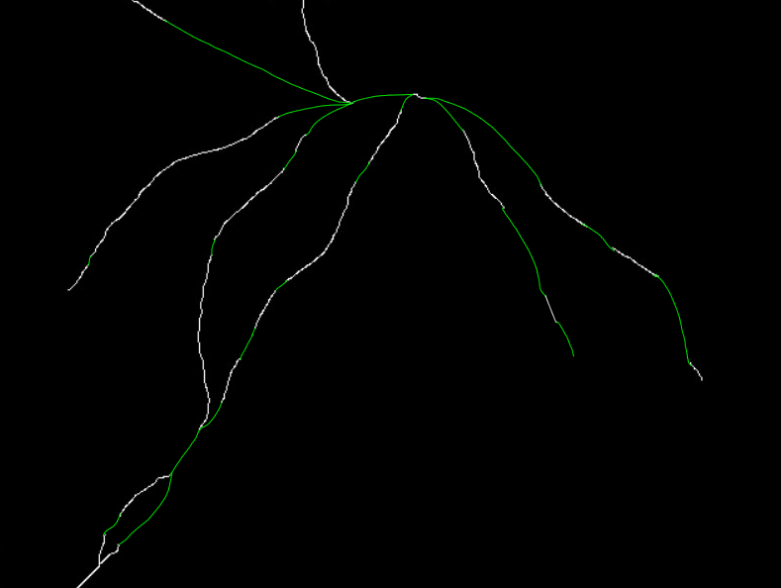

Supplement: Supplementary file 1 — Additional file 1. 16 original root images and the corresponding 16 processed images using the presented algorithm in the paper. [file 13007_2019_518_MOESM1_ESM.zip › test_4.2.png]

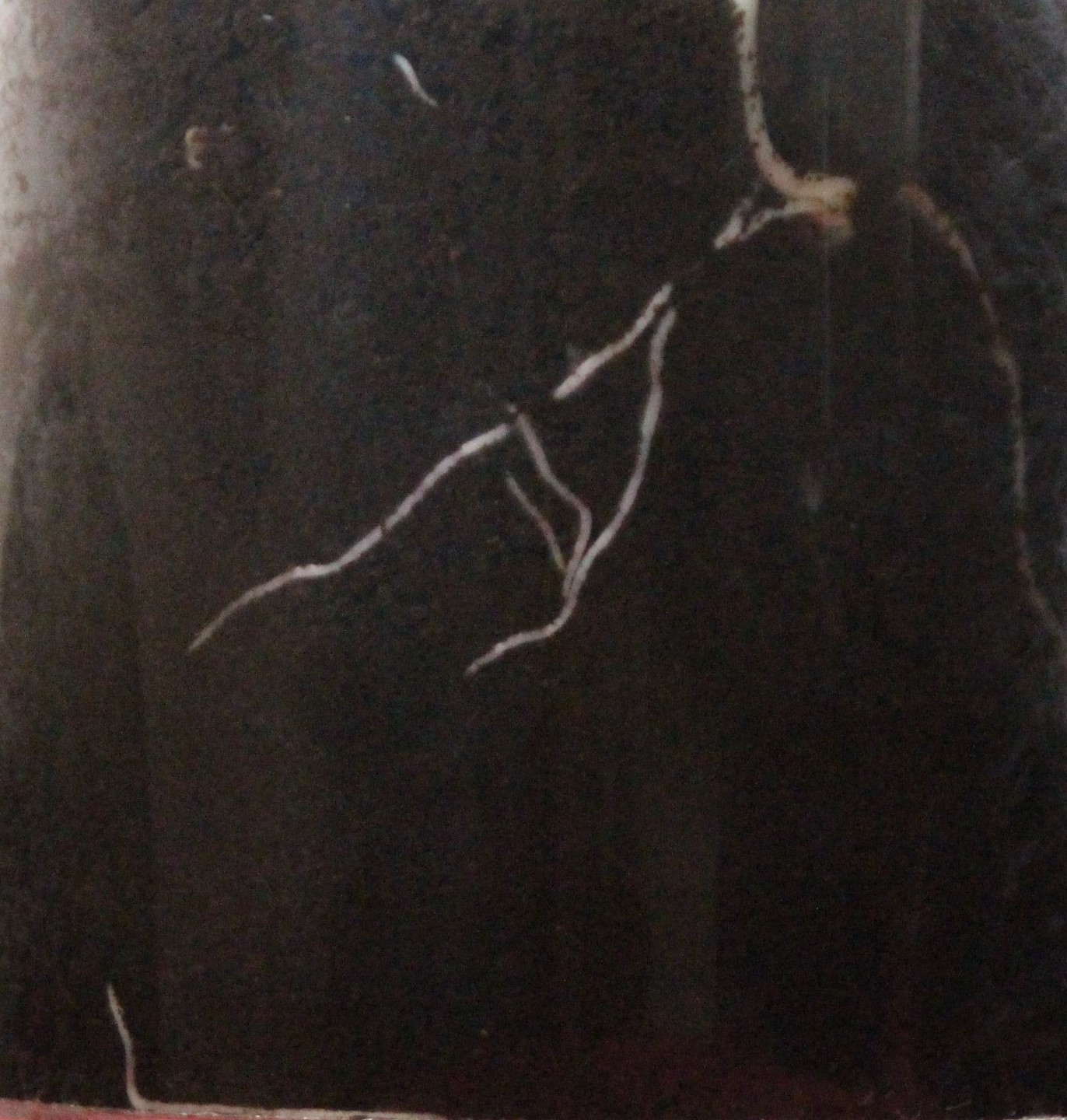

Supplement: Supplementary file 1 — Additional file 1. 16 original root images and the corresponding 16 processed images using the presented algorithm in the paper. [file 13007_2019_518_MOESM1_ESM.zip › test_5.1.JPG]

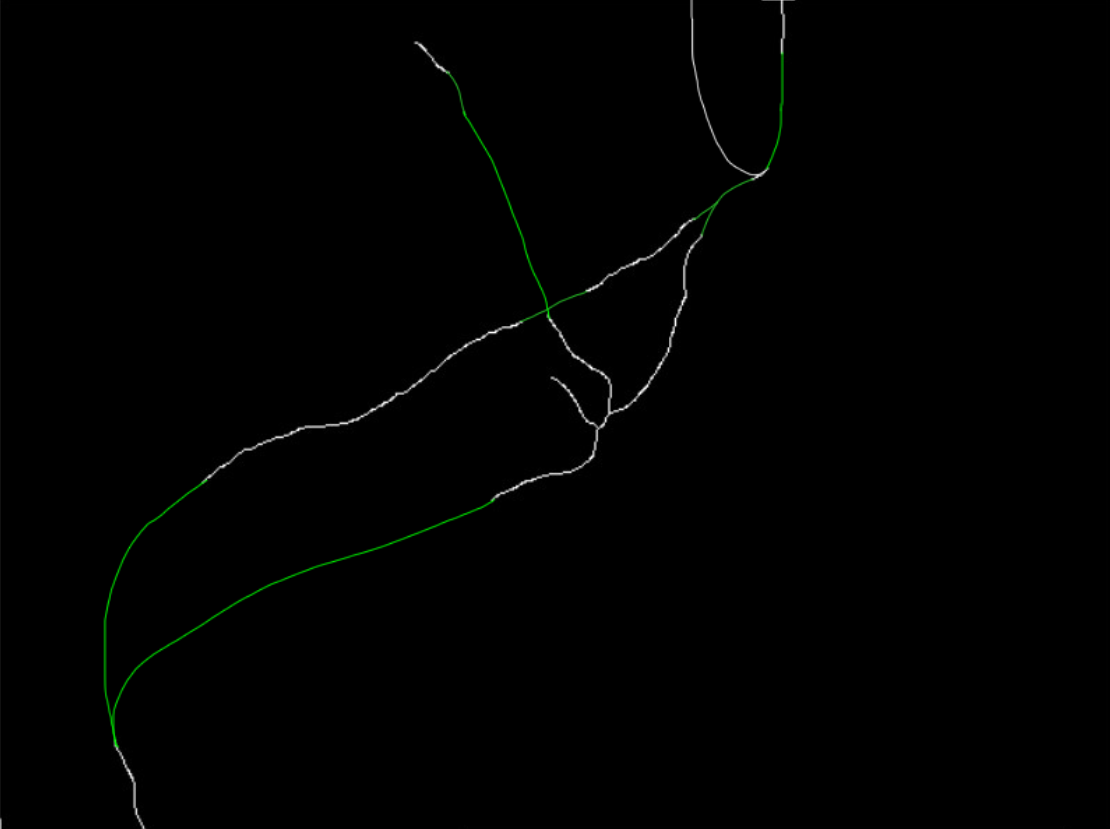

Supplement: Supplementary file 1 — Additional file 1. 16 original root images and the corresponding 16 processed images using the presented algorithm in the paper. [file 13007_2019_518_MOESM1_ESM.zip › test_5.2.png]

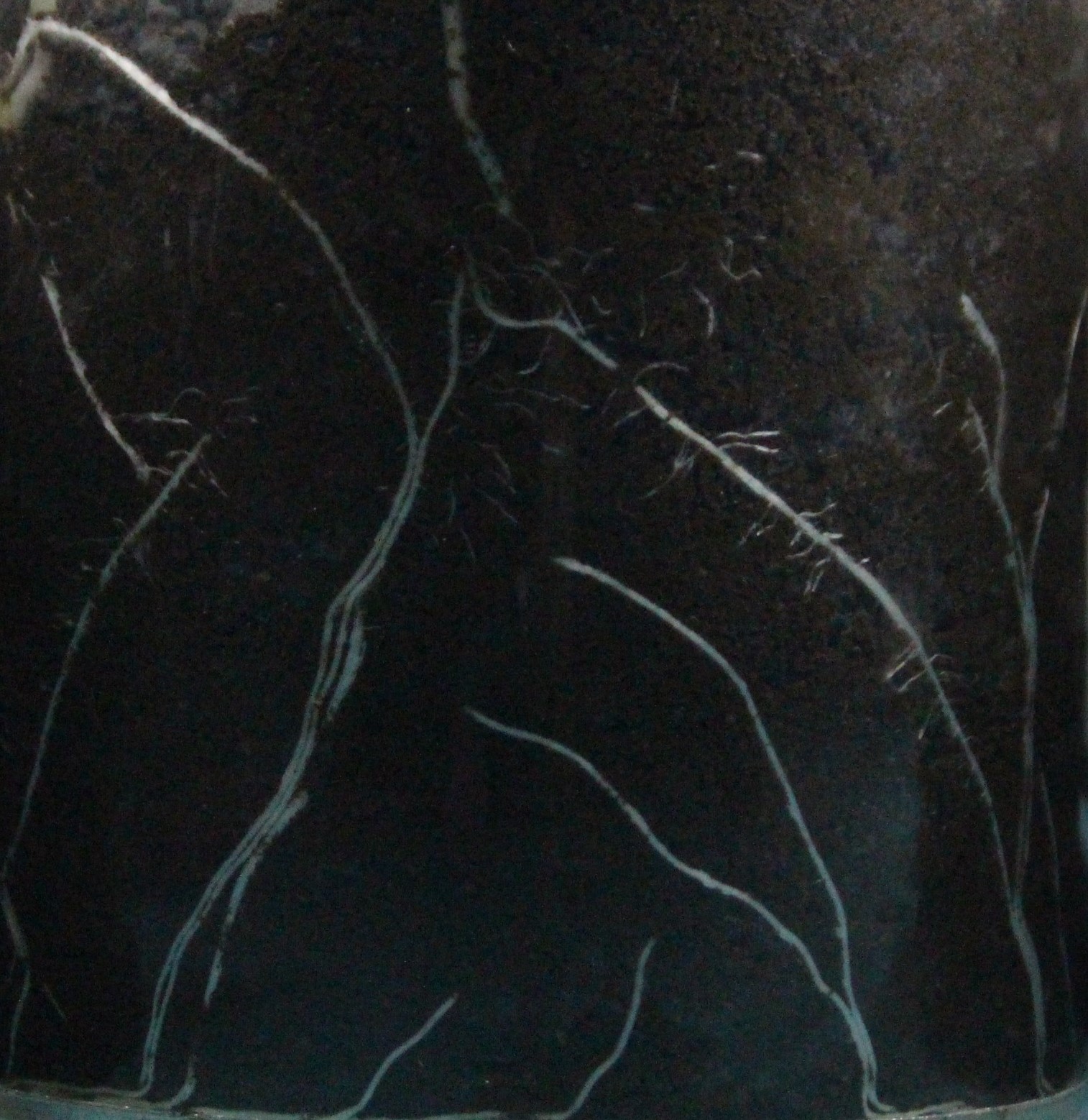

Supplement: Supplementary file 1 — Additional file 1. 16 original root images and the corresponding 16 processed images using the presented algorithm in the paper. [file 13007_2019_518_MOESM1_ESM.zip › test_6.1.JPG]

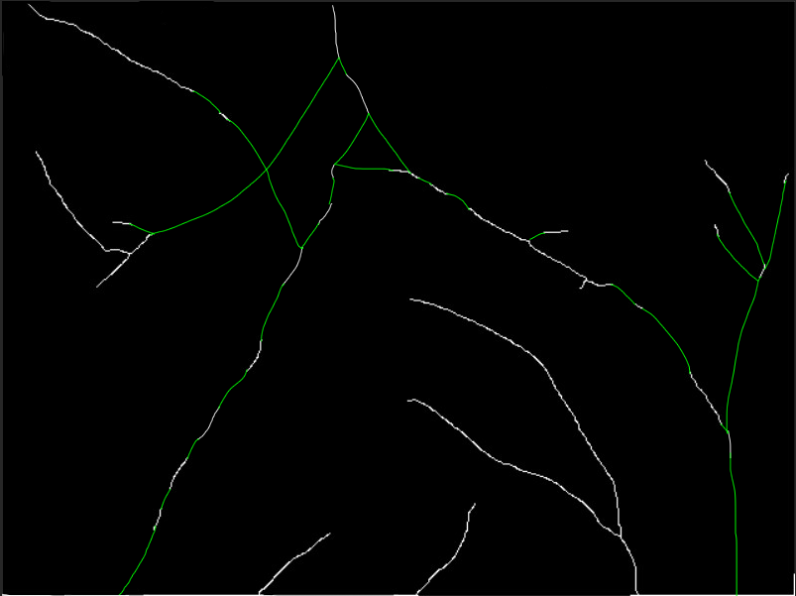

Supplement: Supplementary file 1 — Additional file 1. 16 original root images and the corresponding 16 processed images using the presented algorithm in the paper. [file 13007_2019_518_MOESM1_ESM.zip › test_6.2.png]

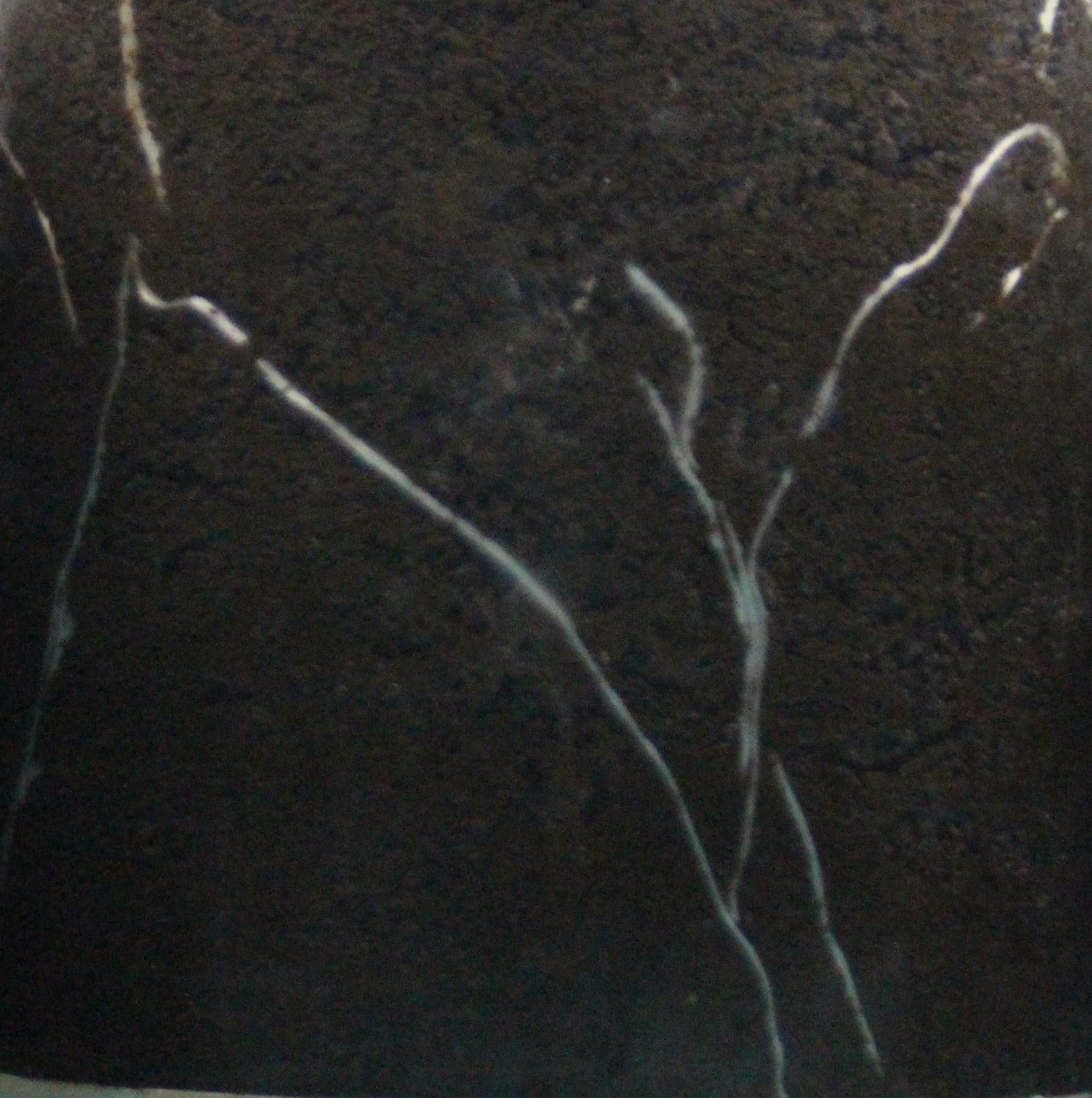

Supplement: Supplementary file 1 — Additional file 1. 16 original root images and the corresponding 16 processed images using the presented algorithm in the paper. [file 13007_2019_518_MOESM1_ESM.zip › test_7.1.JPG]

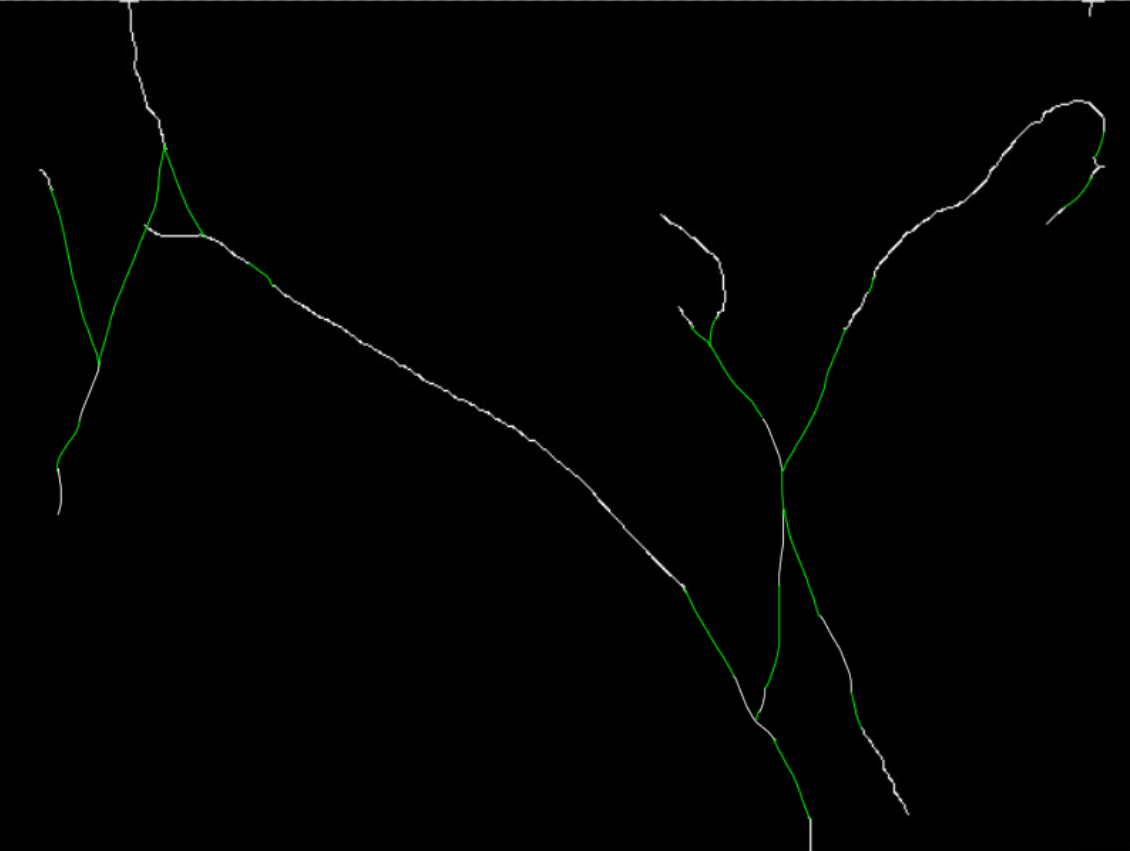

Supplement: Supplementary file 1 — Additional file 1. 16 original root images and the corresponding 16 processed images using the presented algorithm in the paper. [file 13007_2019_518_MOESM1_ESM.zip › test_7.2.png]

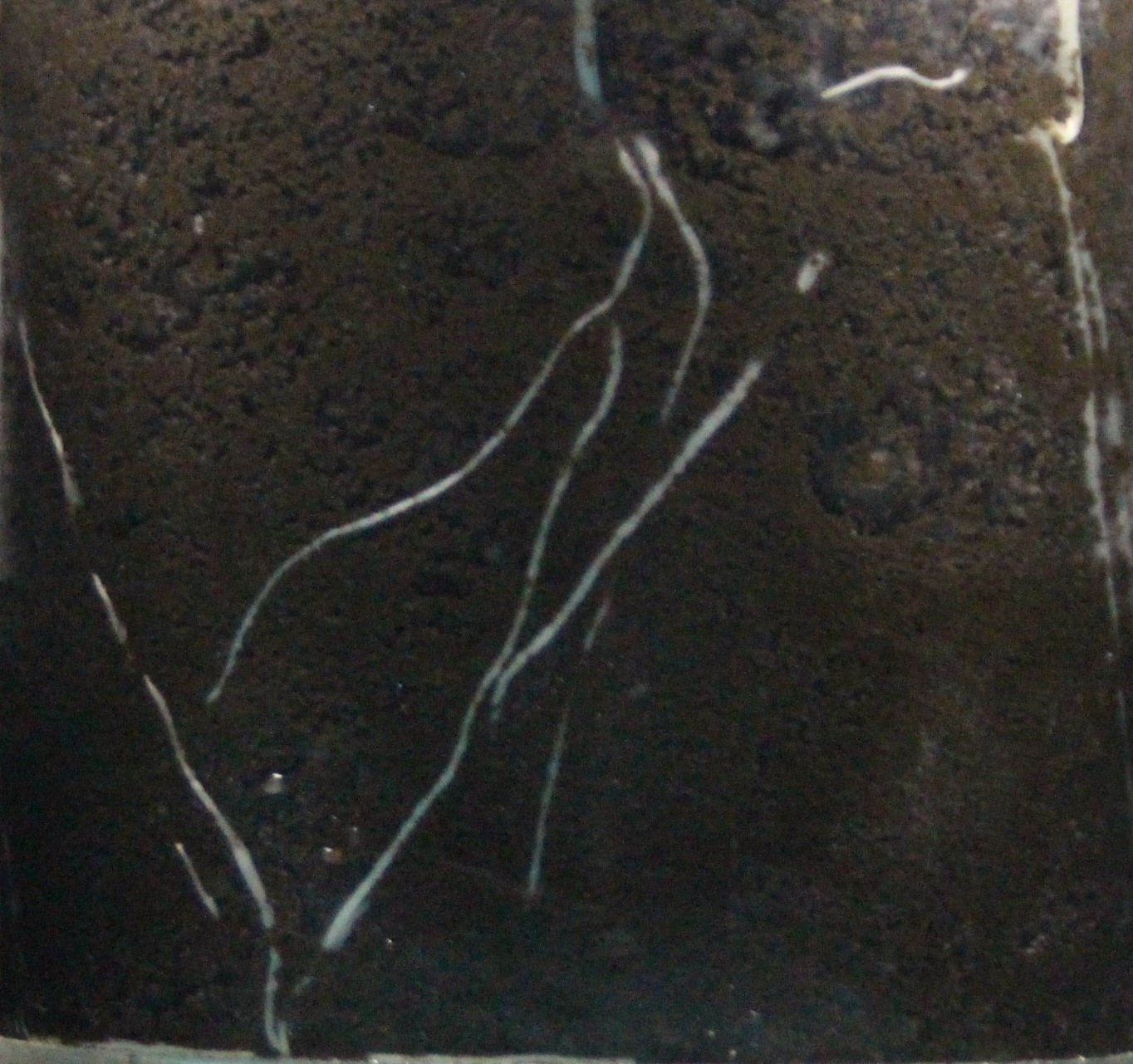

Supplement: Supplementary file 1 — Additional file 1. 16 original root images and the corresponding 16 processed images using the presented algorithm in the paper. [file 13007_2019_518_MOESM1_ESM.zip › test_8.1.JPG]

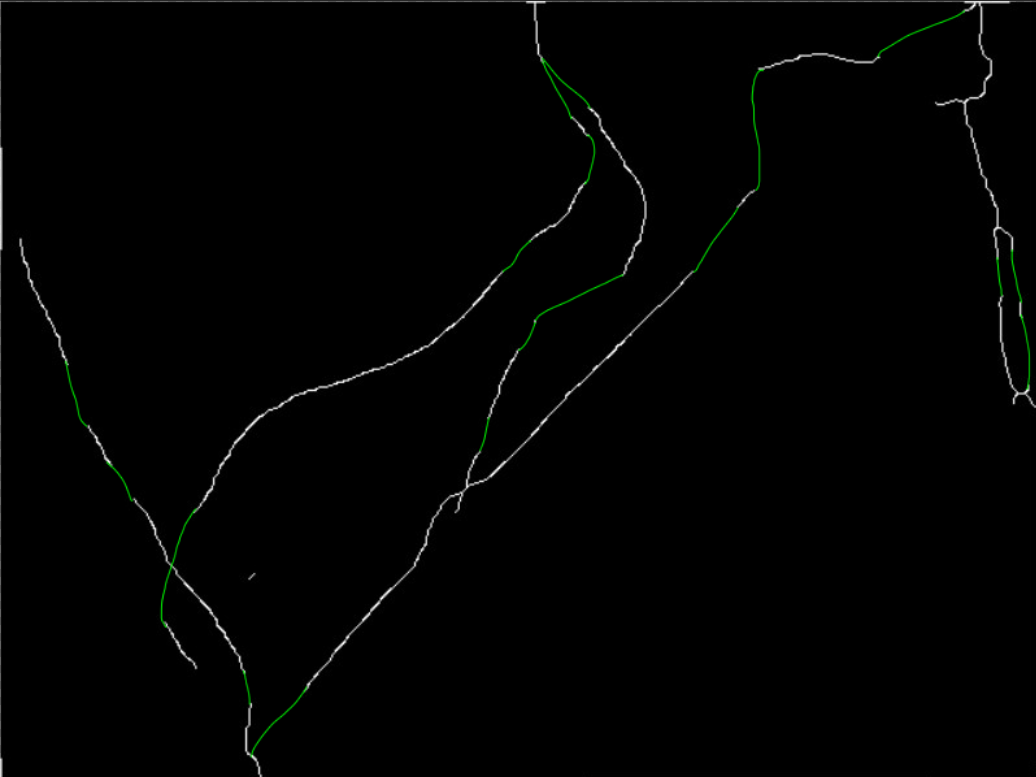

Supplement: Supplementary file 1 — Additional file 1. 16 original root images and the corresponding 16 processed images using the presented algorithm in the paper. [file 13007_2019_518_MOESM1_ESM.zip › test_8.2.png]

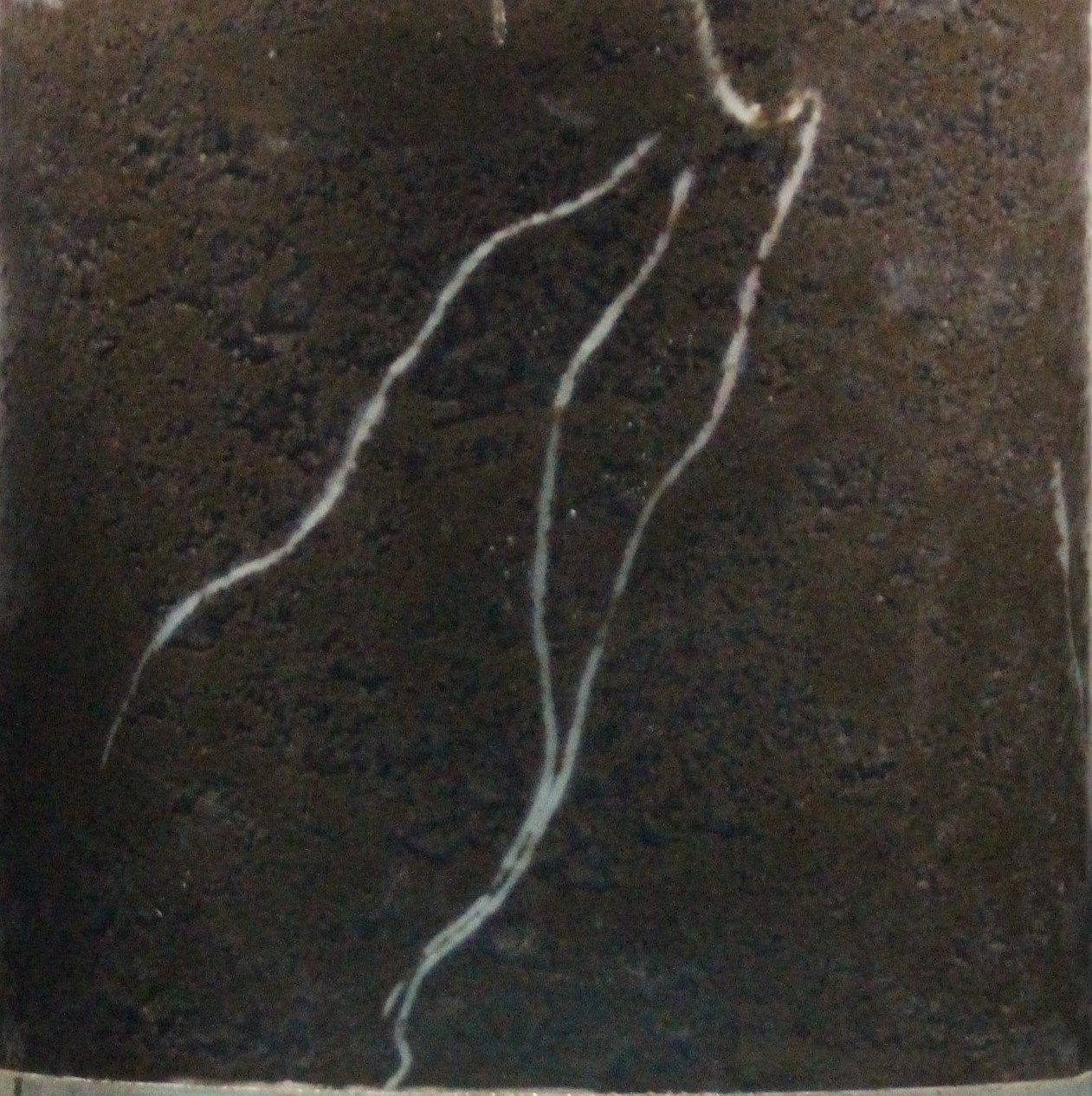

Supplement: Supplementary file 1 — Additional file 1. 16 original root images and the corresponding 16 processed images using the presented algorithm in the paper. [file 13007_2019_518_MOESM1_ESM.zip › test_9.1.JPG]

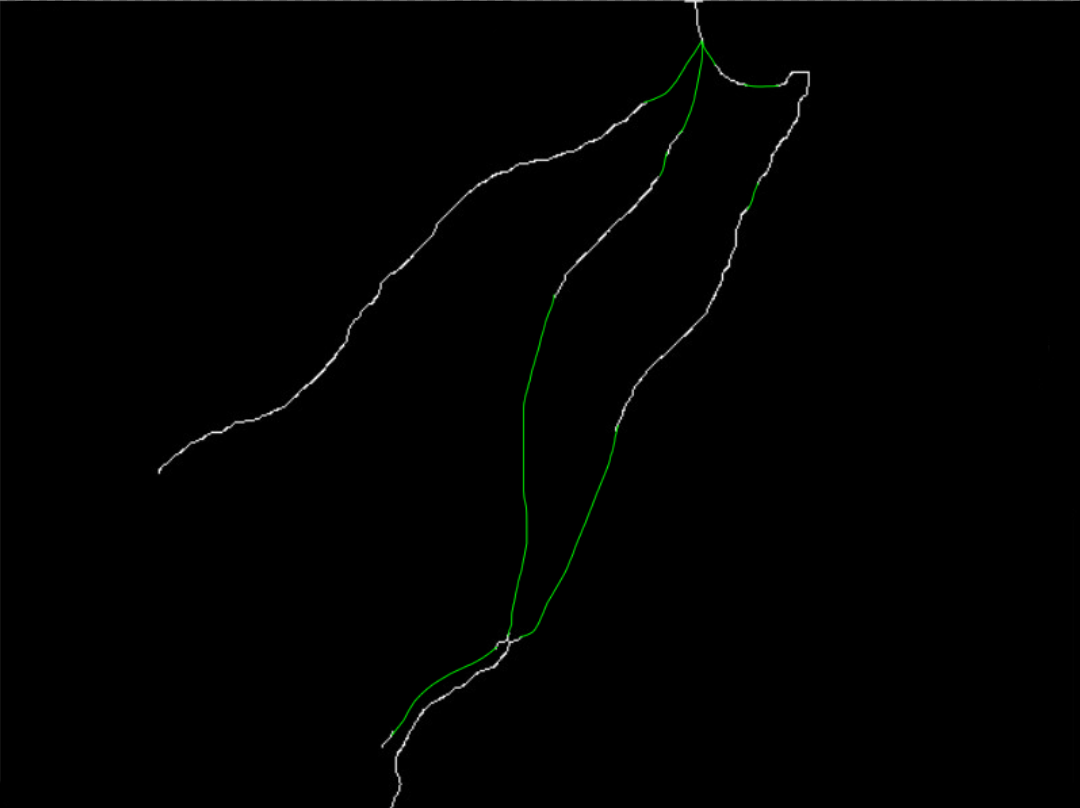

Supplement: Supplementary file 1 — Additional file 1. 16 original root images and the corresponding 16 processed images using the presented algorithm in the paper. [file 13007_2019_518_MOESM1_ESM.zip › test_9.2.png]
